# Tranilast inhibits the expression of genes related to epithelial-mesenchymal transition and angiogenesis in neurofibromin-deficient cells

**DOI:** 10.1038/s41598-018-24484-y

**Published:** 2018-04-17

**Authors:** Ritsuko Harigai, Shigeki Sakai, Hiroyuki Nobusue, Chikako Hirose, Oltea Sampetrean, Noriaki Minami, Yukie Hata, Takashi Kasama, Takanori Hirose, Toshiki Takenouchi, Kenjiro Kosaki, Kazuo Kishi, Hideyuki Saya, Yoshimi Arima

**Affiliations:** 1 Division of Gene Regulation, Institute for Advanced Medical Research, Keio University School of Medicine, Tokyo, 160-8582 Japan; 2 Department of Plastic and Reconstructive Surgery, Keio University School of Medicine, Tokyo, 160-8582 Japan; 3 Department of Surgery, Keio University School of Medicine, Tokyo, 160-8582 Japan; 4 Department of Neurosurgery, Kobe University Graduate School of Medicine, Hyogo, 650-0017 Japan; 5 Department of Biomedical Research & Development, Link Genomics Inc, Tokyo, 103-0024 Japan; 6 Department of Pathology for Regional Communication, Kobe University Graduate School of Medicine, Hyogo, 650-0017 Japan; 7 Department of Paediatrics, Keio University School of Medicine, Tokyo, 160-8582 Japan; 8 Center for Medical Genetics, Keio University School of Medicine, Tokyo, 160-8582 Japan

## Abstract

Neurofibromatosis type 1 (NF1) is caused by germline mutations in the *NF1* gene and is characterized by café au lait spots and benign tumours known as neurofibromas. *NF1* encodes the tumour suppressor protein neurofibromin, which negatively regulates the small GTPase Ras, with the constitutive activation of Ras signalling resulting from *NF1* mutations being thought to underlie neurofibroma development. We previously showed that knockdown of neurofibromin triggers epithelial-mesenchymal transition (EMT) signalling and that such signalling is activated in NF1-associated neurofibromas. With the use of a cell-based drug screening assay, we have now identified the antiallergy drug tranilast (*N*-(3,4-dimethoxycinnamoyl) anthranilic acid) as an inhibitor of EMT and found that it attenuated the expression of mesenchymal markers and angiogenesis-related genes in *NF1*-mutated sNF96.2 cells and in neurofibroma cells from NF1 patients. Tranilast also suppressed the proliferation of neurofibromin-deficient cells *in vitro* more effectively than it did that of intact cells. In addition, tranilast inhibited sNF96.2 cell migration and proliferation *in vivo*. Knockdown of type III collagen (COL3A1) also suppressed the proliferation of neurofibroma cells, whereas expression of *COL3A1* and *SOX2* was increased in tranilast-resistant cells, suggesting that COL3A1 and the transcription factor SOX2 might contribute to the development of tranilast resistance.

## Introduction

The *NF1* tumour suppressor gene is located at human chromosomal region 17q11.2 and encodes a 2818–amino acid protein termed neurofibromin^1^. Neurofibromin, which is localized to the cytoplasm of numerous cell types, contains a mammalian Ras GTPase-activating protein (RasGAP) domain that negatively regulates the Ras signalling pathway by promoting the conversion of GTP-bound (active) Ras to the GDP-bound (inactive) state. Loss or mutation of neurofibromin thus results in hyperactivation of Ras and engagement of its downstream effectors^2,3^.

Mutations of the *NF1* gene give rise to neurofibromatosis type 1 (NF1), also known as von Recklinghausen disease^4^. NF1 is an autosomal dominant inherited disease that affects one in ~3000 live births, with 30% to 50% of *NF1* mutations arising *de novo*. Individuals with NF1 manifest a variety of clinical symptoms including peripheral and central nervous system tumours, pigmentary changes such as multiple café au lait spots, bone defects, cardiovascular abnormalities, and learning disabilities. The most common symptom of NF1 is the development of benign peripheral nerve sheath tumours known as neurofibromas. In addition, patients with NF1 are at an increased risk for the development of leukaemia, rhabdomyosarcoma, pheochromocytoma, gastrointestinal stromal tumours, early-onset breast cancer, and high-grade malignant glioblastoma^5,6^. Various clinical trials of molecularly targeted drugs are under way for NF1, with the results of phase II trials of several agents including imatinib, tipifarnib, pirfenidone, and sirolimus for the treatment of plexiform neurofibromas of NF1 patients having been presented^7–11^. At the present time, however, there are no effective therapeutic agents for benign neurofibromas.

The epithelial-mesenchymal transition (EMT) is defined by the loss of epithelial characteristics and the acquisition of mesenchymal phenotypes and is mediated by activation of EMT transcription factors (EMT-TFs) such as Snail, Slug, Twist, ZEB1, and ZEB2 (ref.^12^). A key molecular feature of EMT is down-regulation of E-cadherin, a cell adhesion molecule present in the plasma membrane of most normal epithelial cells. However, recent studies have shown that EMT signalling is also activated in nonepithelial tumours, including gliomas, hematopoietic malignancies, and sarcomas, and that it plays an important role in disease progression and prognosis^13^. We have previously shown that EMT-TFs are activated in neurofibromin-depleted cells and in NF1-associated neurofibroma specimens^14^. EMT is also associated with increased production of extracellular matrix (ECM) components by cells as well as with an enhanced migratory capacity and resistance to apoptosis, suggesting that EMT signals might serve as therapeutic targets for the treatment of NF1-associated neurofibromas.

To identify inhibitors of EMT, we performed a drug screening assay based on focus formation. We thereby identified tranilast (*N*-(3,4-dimethoxycinnamoyl) anthranilic acid) as an inhibitor of EMT. Tranilast is an antiallergy drug that is also administered for the treatment of keloids and hypertrophic scars. In the present study, we examined the effects of tranilast on neurofibromin-deficient cell lines as well as on neurofibroma cells derived from NF1 patients.

## Results

### Tranilast inhibits TNF-α– and TGF-β2–induced aggregation of ARPE-19 cells

**Figure 1:**
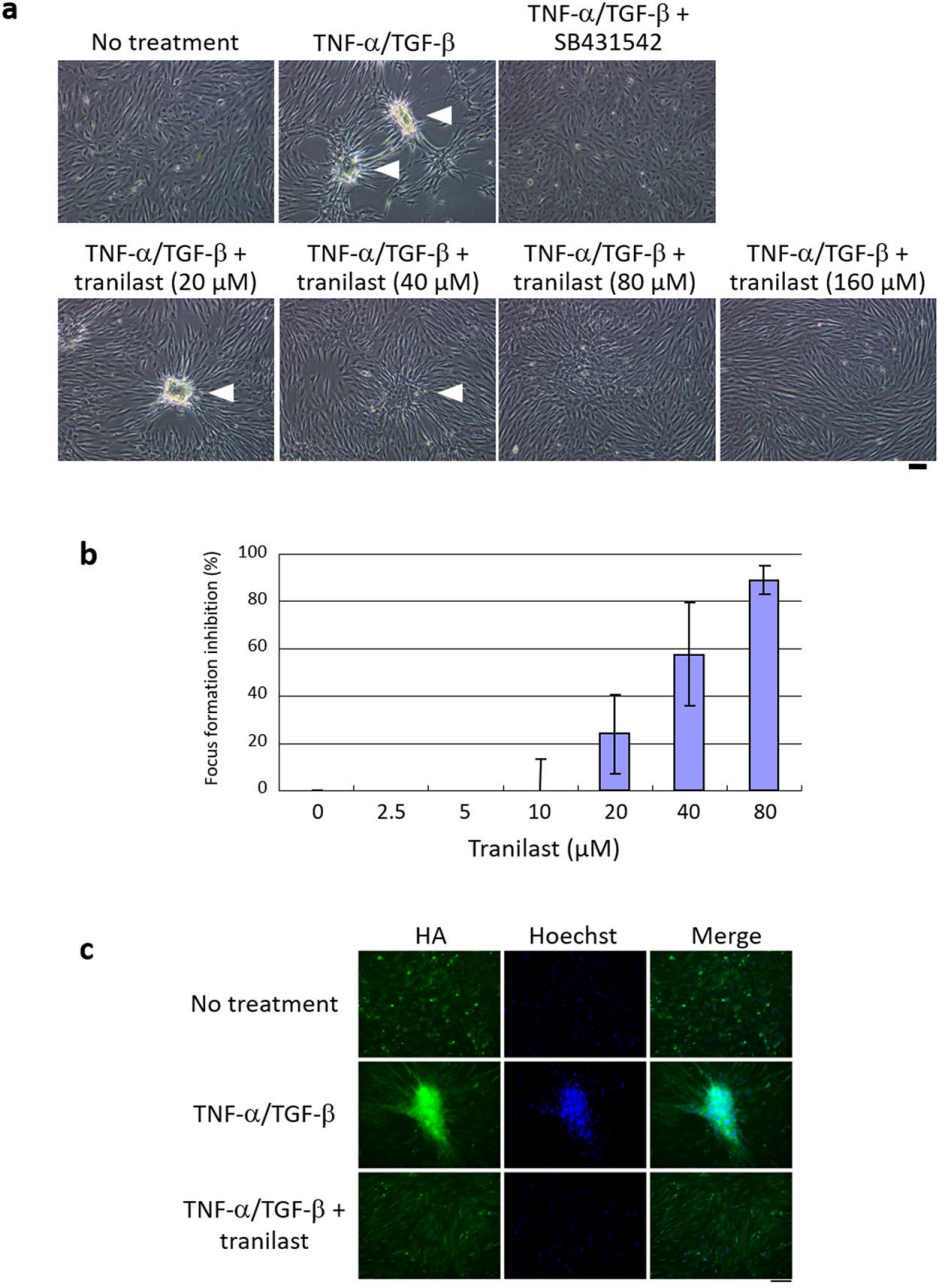
Tranilast inhibits EMT induced by TNF-α and TGF-β2 in ARPE-19 cells. (**a**) Phase-contrast microscopy of ARPE-19 cells that had been cultured for 5 days to 100% confluence and then incubated for 2 days in the absence or presence of TNF-α (100 ng/ml) and TGF-β2 (5 ng/ml) as well as of SB431542 (10 µM) or tranilast (20, 40, 80, or 160 µM). Arrowheads indicate cell aggregation. Scale bar, 100 µm. (**b**) Concentration-dependent inhibition by tranilast of ARPE-19 focus formation induced by TNF-α and TGF-β2 was assayed as in a. Data are means ± s.d. from three independent experiments. (**c**) ARPE-19 cells incubated in the absence or presence of TNF-α/TGF-β2 and tranilast (160 µM) for 2 days were stained with biotinylated HABP (and Alexa Fluor 488–conjugated streptavidin) for detection of HA as well as with Hoechst 33342 for detection of nuclei. The cells were then observed by fluorescence microscopy. Scale bar, 100 µm.

We developed a cell-based screening assay to identify inhibitors of EMT. We previously showed that the combination of tumour necrosis factor–α (TNF-α) and transforming growth factor–β2 (TGF-β2) activates EMT signalling and induces cell aggregation in the human retinal pigment epithelial cell line ARPE-19 (ref.^15^). This cell aggregation, or focus formation, involves production of ECM components including collagen and hyaluronic acid (HA) and is a characteristic feature of EMT activation^16–20^. We thus examined focus formation by ARPE-19 cells as an indicator of EMT. Focus formation induced by TNF-α and TGF-β2 was inhibited by exposure of the cells to the TGF receptor inhibitor SB431542, which was tested as a positive control (Fig. 1a). Compounds that inhibited focus formation by ≥20% with minimal cytotoxicity at a concentration of 20 μM were considered positive. Screening of >1500 compounds approved by the U.S. Food and Drug Administration identified eight drugs that were able to inhibit TNF-α/TGF-β2–induced focus formation. One of these putative EMT inhibitors was tranilast, an antiallergy drug that is also used for the treatment of inflammatory conditions and keloids^21,22^. Tranilast thus inhibited focus formation at a concentration of 20 µM (Fig. 1a), with the median inhibitory concentration being 35.86 ± 17.78 µM (Fig. 1b). We also examined the effect of tranilast on HA production, which is induced by EMT signals^23–26^. Fluorescence microscopic analysis of cells stained with biotinylated HA binding protein (HABP) revealed that tranilast inhibited the production of HA induced by TNF-α/TGF-β2 (Fig. 1c). These data thus suggested that tranilast inhibits EMT induced by TNF-α and TGF-β2 in ARPE-19 cells.

### Tranilast suppresses EMT characteristics in neurofibromin-deficient cells

Depletion of neurofibromin in HeLa human epithelial carcinoma cells by transfection with a specific small interfering RNA (siRNA) induced a change in cell morphology from cuboidal to spindle-shaped, with reverse transcription (RT) and real-time polymerase chain reaction (PCR) analysis also revealing that depletion of neurofibromin increased the expression of mesenchymal marker genes including those encoding fibronectin (*FN1*) and N-cadherin (*CDH2*) as well as that of those for the EMT-TFs Slug (*SNAI2*) and Twist (*TWIST1*) (Supplementary Fig. S1). These results confirmed our previous observations that deficiency of neurofibromin is associated with EMT signalling^14^.

**Figure 2:**
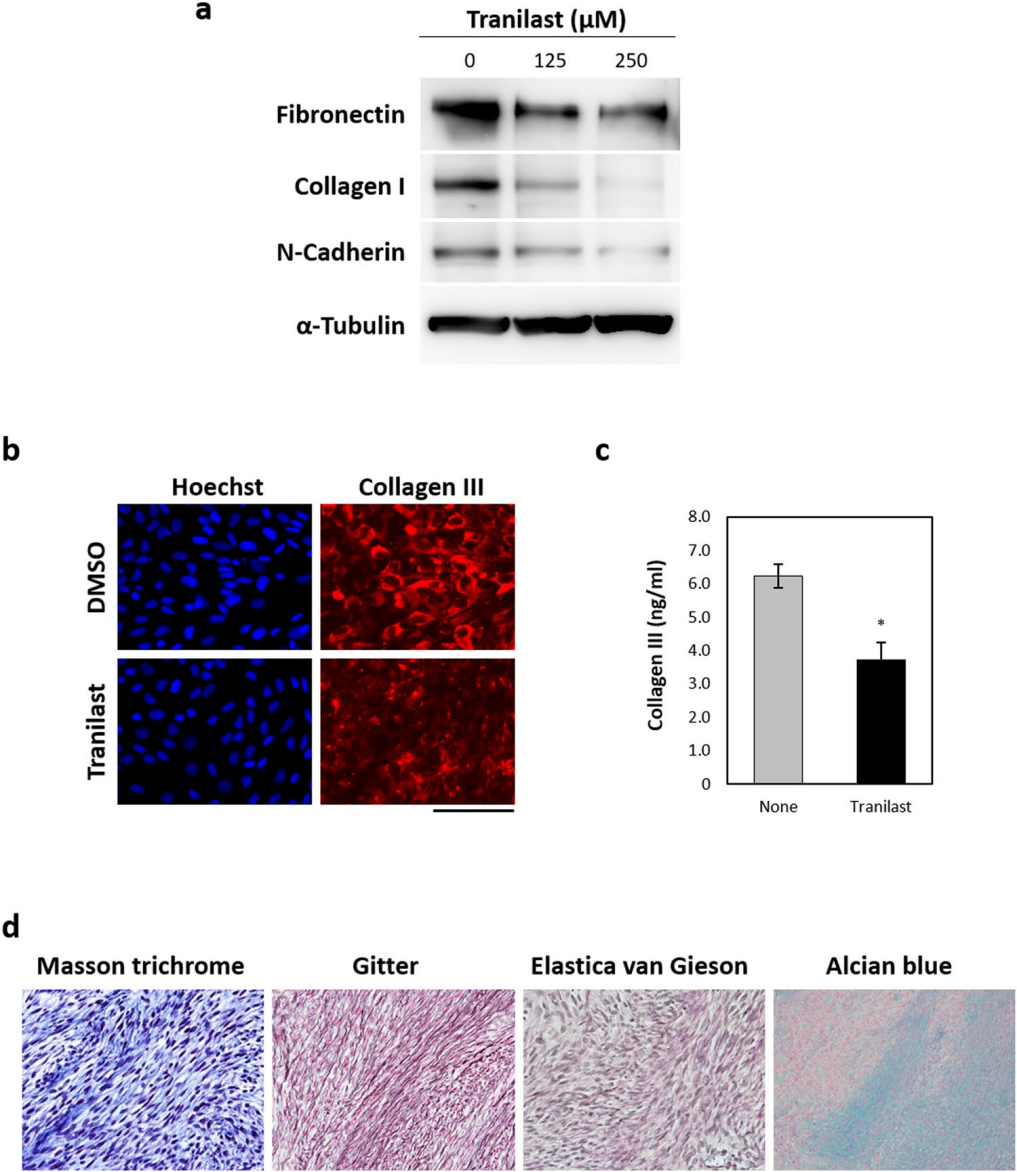
Tranilast attenuates the expression of mesenchymal markers in sNF96.2 cells. (**a**) Immunoblot analysis of fibronectin, collagen type I, N-cadherin, and α-tubulin (loading control) in sNF96.2 cells treated with the indicated concentrations of tranilast for 48 h. Blots are derived from different regions of the same gel. Uncropped images are shown in Supplementary Fig. S6. (**b**) Immunofluorescence analysis of collagen type III in sNF96.2 cells treated with tranilast (250 µM) or dimethyl sulfoxide (DMSO) vehicle for 48 h. Nuclei were stained with Hoechst 33342. Scale bar, 100 µm. (**c**) Quantification by ELISA of collagen type III in sNF96.2 cells incubated with or without tranilast (250 µM) for 48 h. Cell lysates were assayed. Data are means ± s.d. for duplicates from a representative experiment. *P < 0.05 versus control (Student’s unpaired *t* test). (**d**) Tumours formed by injected sNF96.2 cells in the brain of NOD/SCID recipient mice were subjected to histological analysis by Masson’s trichrome, Gitter, Elastica van Gieson, and Alcian blue staining.

We next examined the effects of tranilast on the *NF1*-mutated human Schwann-like cell line sNF96.2. Immunoblot analysis revealed that expression of mesenchymal markers—including fibronectin, collagen type I, and N-cadherin—was decreased by treatment of these cells with tranilast (Fig. 2a). Quantitative RT-PCR analysis also showed that expression of the genes for various EMT-TFs, collagens, hyaluronan synthases, and integrins in these cells was inhibited by tranilast (Supplementary Fig. S2). Furthermore, tranilast attenuated in particular the expression of collagen type III in sNF96.2 cells as revealed by both immunofluorescence analysis (Fig. 2b) and an enzyme-linked immunosorbent assay (ELISA) (Fig. 2c). These results suggested that tranilast suppresses the mesenchymal phenotypes of sNF96.2 cells including the expression of various ECM components.

We subjected tumours formed by sNF96.2 cells in the brain of NOD/SCID recipient mice to histological analysis by Masson’s trichrome staining for collagen fibres including collagen type I, Gitter staining for reticular fibres including collagen type III, Elastica van Gieson staining for elastic fibres, and Alcian blue staining for acid mucosubstances and acidic mucins (Fig. 2d). Small collagen fibres rendered dark blue by Masson’s trichrome stain were detected between the tumour cells, and abundant thick reticular fibres rendered black by Gitter staining were also apparent surrounding the tumour cells. Elastic fibres (black staining) were not detected by Elastica van Gieson staining, whereas Alcian blue–positive material was observed in regions of low cell density.

We previously showed that EMT-TFs including ZEB1 are activated in neurofibromin-depleted cells and in NF1-associated neurofibroma specimens^14^. We further examined whether the mesenchymal marker vimentin and EMT-related collagens are also expressed in NF1-associated neurofibroma specimens. Immunohistochemical analysis of formalin-fixed tissue samples from two patients revealed the expression of ZEB1, vimentin, collagen type I, collagen type III, and SOX2 (Supplementary Fig. S3). These results thus supported the notion that EMT signals might be therapeutic targets for the treatment of NF1-associated neurofibromas.

### Tranilast suppresses the proliferation of *NF1*-mutated cells *in vitro*

**Figure 3:**
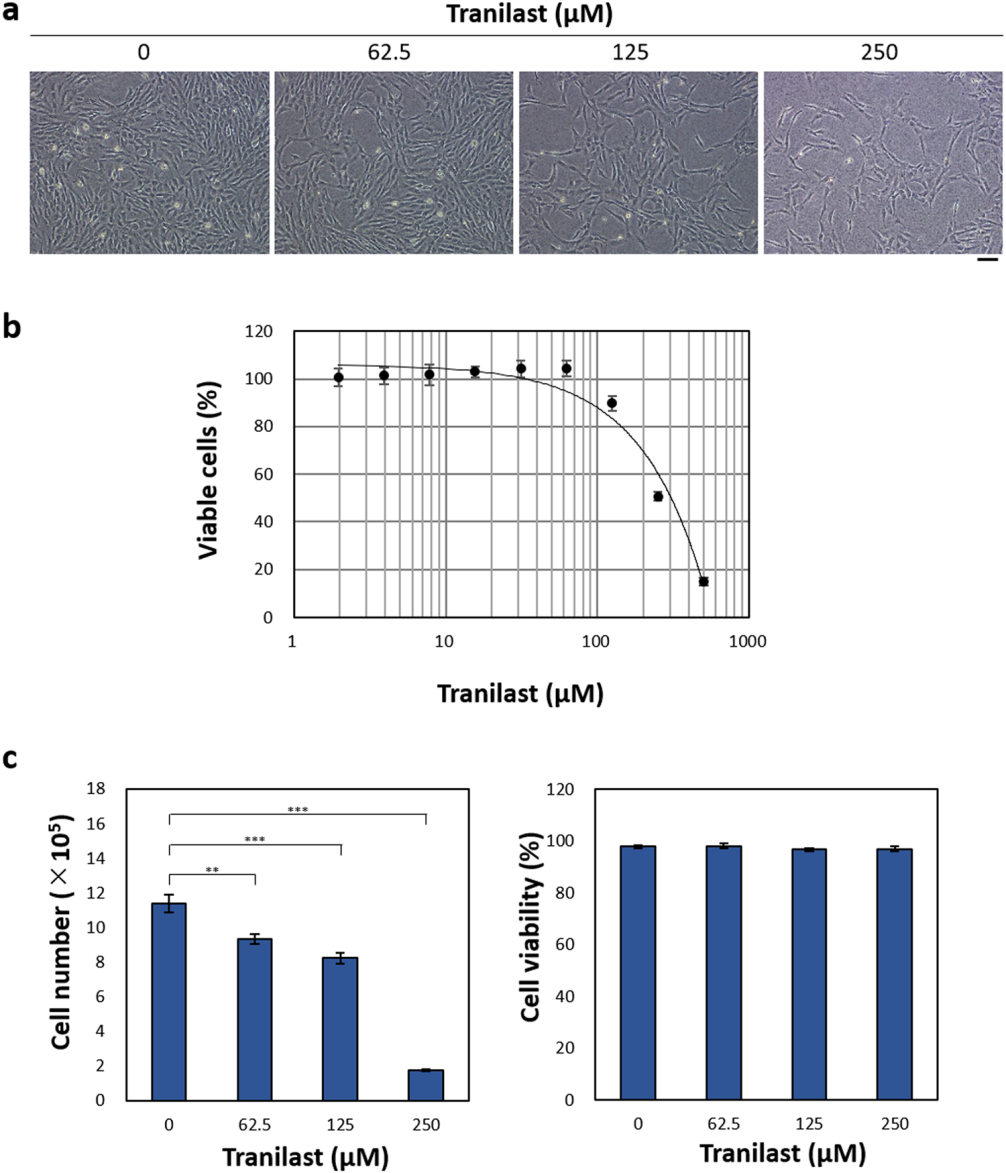
Tranilast inhibits sNF96.2 cell proliferation. (**a**) Phase-contrast microscopy of sNF96.2 cells treated with the indicated concentrations of tranilast for 48 h. Scale bar, 100 µm. (**b**) Concentration-response curve for the inhibition of sNF96.2 cell proliferation determined by measurement of the number of viable cells with the CellTiter-Glo (Promega) assay after exposure of the cells to the drug for 48 h. Data are means ± s.d. for six replicates from a representative experiment. (**c**) sNF96.2 cells were treated with the indicated concentrations of tranilast for 48 h, after which the number of viable cells and the percentage of viable cells were determined on the basis of trypan blue exclusion. Data are means ± s.d. for triplicates from a representative experiment. **P < 0.01 (Student’s unpaired *t* test).

We examined the effect of tranilast on the proliferation of sNF96.2 cells *in vitro*. Exposure of the cells to tranilast for 48 h resulted in a concentration-dependent reduction in the number of viable cells compared with that for control cultures incubated in the absence of the drug (Fig. 3). Given that tranilast did not appear to induce cell death, these data indicated that tranilast inhibits the proliferation of sNF96.2 cells. The median inhibitory concentration of tranilast for this effect was >200 µM, a concentration that appears to be achievable clinically given that the administration of tranilast at 200 mg three times a day for 7 days in humans was previously estimated to result in a maximum plasma concentration of 73.0 μg/ml, which corresponds to 223 μM^27^.

**Figure 4:**
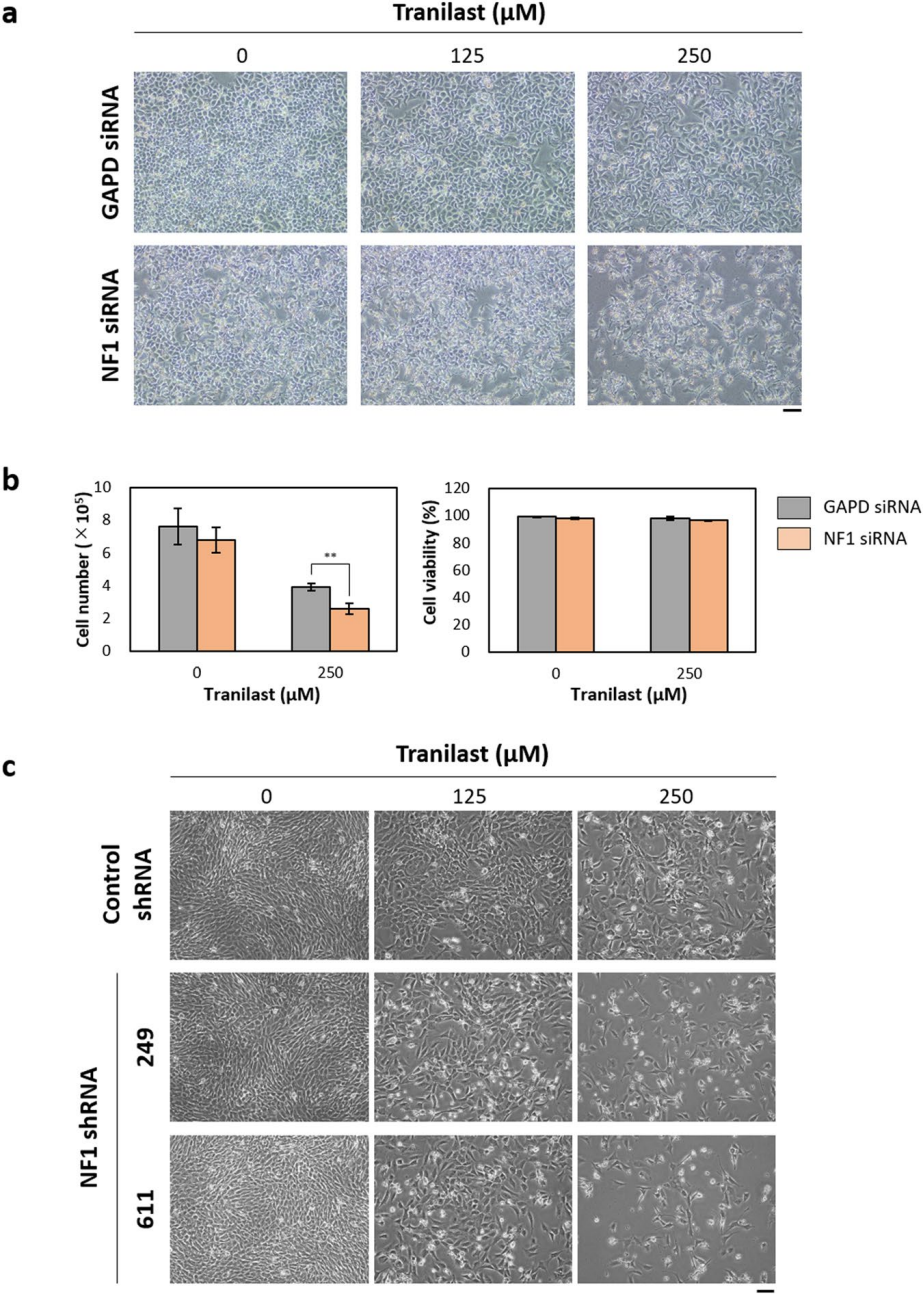
Neurofibromin depletion confers sensitivity to tranilast. (**a**) HeLa cells were transfected with control (GAPD) or neurofibromin (NF1) siRNAs for 1 day, exposed to various concentrations of tranilast for 48 h, and then examined by phase-contrast microscopy. Scale bar, 100 µm. (**b**) HeLa cells transfected with GAPD or NF1 siRNAs as in **a** were incubated in the absence or presence of tranilast (250 µM) for 48 h, after which the number of viable cells and the percentage of viable cells were measured on the basis of trypan blue exclusion. Data are means ± s.d. for triplicates from a representative experiment. **P < 0.01 (Student’s unpaired *t* test). (**c**) NIH3T3 cells stably transfected with plasmids for a negative control shRNA or either of two NF1 shRNAs (249 or 611) were incubated with the indicated concentrations of tranilast for 48 h and then examined by phase-contrast microscopy. Scale bar, 100 µm.

To clarify the relation between neurofibromin deficiency and tranilast sensitivity, we examined the effects of the drug on HeLa cells transfected with control or neurofibromin siRNAs. Tranilast inhibited the proliferation of HeLa cells depleted of neurofibromin to a much greater extent than it did that of the control cells (Fig. 4a,b). We also found that tranilast suppressed the growth of NIH3T3 mouse embryonic fibroblasts expressing either of two short hairpin RNAs (shRNAs) specific for *NF1* mRNA to a greater extent than it did that of those expressing a control shRNA (Fig. 4c). These data suggested that loss of *NF1* expression is directly related to tranilast sensitivity.

### Tranilast attenuates the expression of angiogenesis-related genes

**Figure 5:**
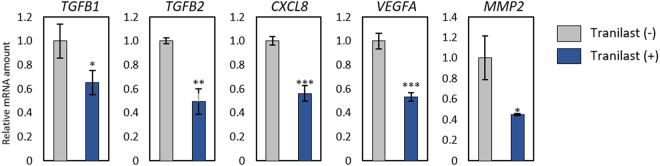
Tranilast suppresses the expression of angiogenesis-related genes in sNF96.2 cells. sNF96.2 cells that had been incubated in the absence or presence of tranilast (250 μM) for 48 h were subjected to quantitative RT-PCR analysis of TGF-β1, TGF-β2, IL-8 (CXCL8), VEGF-A, and MMP2 mRNAs. Data are means ± s.d. for triplicates from a representative experiment. *P < 0.05, **P < 0.01, ***P < 0.001 versus corresponding control value (Student’s unpaired *t* test).

Like normal organs, tumours require a blood supply to satisfy their demands for oxygen and nutrients as well as to accomplish other metabolic functions. Angiogenesis, the process by which new blood vessels develop from a pre-existing vascular network, is regulated by cancer cells and by components of the tumour microenvironment including tumour-associated stromal cells, cytokines, growth factors, ECM, and secreted microvesicles^28^. We found that tranilast down-regulated the abundance of mRNAs for TGF-β, interleukin (IL)–8, vascular endothelial growth factor (VEGF), and matrix metalloproteinase 2 (MMP2) in sNF96.2 cells (Fig. 5). All of these factors are thought to promote angiogenesis and have been found to be associated with tumour angiogenesis^29–32^. These results thus suggested the possibility that tranilast might suppress tumour progression in NF1 patients.

The expression of TGF-β, IL-8, VEGF, and MMP2 genes was increased in sNF96.2 cells compared with normal human Schwann cells (HSCs) (Supplementary Fig. S4). However, transient depletion of neurofibromin by siRNA transfection did not significantly increase the expression of these genes in HSCs (Supplementary Fig. S4). These results suggested that chronic deficiency of neurofibromin may be indirectly related to angiogenesis.

### Tranilast suppresses invasion and proliferation in *NF1*-mutated tumour cells

**Figure 6:**
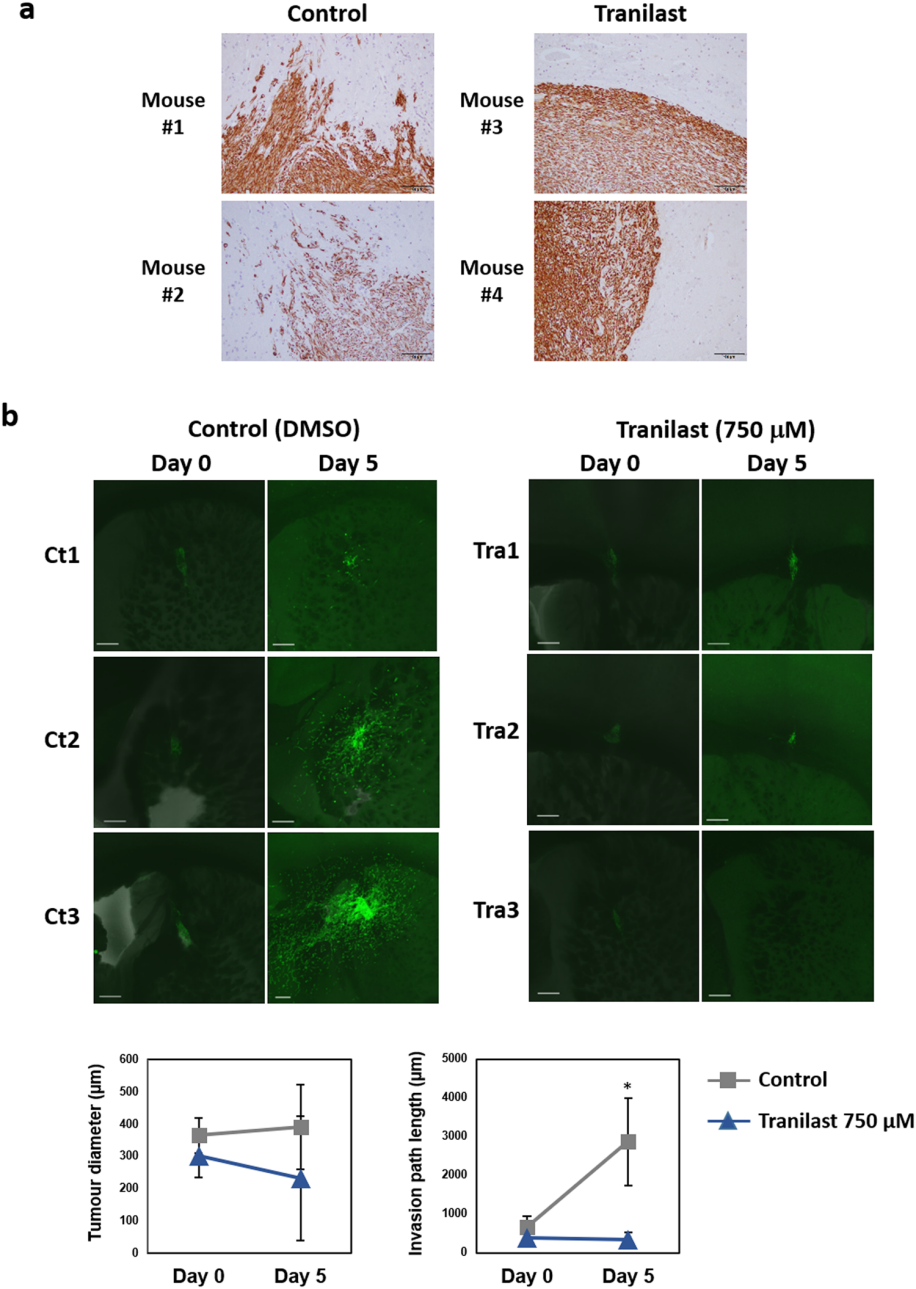
Tranilast inhibits sNF96.2 cell proliferation and invasion in brain tissue. (**a**) sNF96.2 cells were injected into the brain of NOD/SCID mice. After 3 weeks, the mice were treated orally with tranilast (300 mg/kg) or vehicle once a day for 8 weeks. The brain was then removed from four representative mice, and formalin-fixed, paraffin-embedded sections of the xenograft tumours were subjected to immunohistochemical analysis with antibodies to vimentin. Scale bars, 100 µm. (**b**) sNF96.2-GFP cells were injected into the brain of NOD/SCID mice. After 6 weeks, the brain was removed and thin slices of brain tissue containing tumour cells were cultured in medium containing tranilast (750 µM) or DMSO vehicle for 0 or 5 days, at which times the same areas (Ct1–3 and Tra1–3) were examined by confocal fluorescence microscopy and photographed. Scale bars, 300 µm. Maximum tumour diameter and maximum length of the invasion path were determined from the images. Data are means ± s.d. (*n* = 3). *P < 0.05 versus corresponding tranilast value (Student’s unpaired *t* test).

Our results suggested that tranilast inhibits EMT-like changes and angiogenesis-related gene expression and shows potential antitumour activity for *NF1*-mutated tumour cells. To test this scenario further, we again transplanted sNF96.2 cells into the brain of NOD/SCID mice and allowed the cells to form tumours. After 3 weeks, the mice were treated orally once a day for 8 weeks with tranilast (300 mg/kg) or vehicle. Immunohistochemical analysis revealed that tranilast treatment markedly inhibited migration of the vimentin-positive tumour cells into the host brain tissue (Fig. 6a), suggesting that tranilast suppresses sNF96.2 cell invasion *in vivo*. Although the sNF96.2 cell line was derived from a recurrent mass in an NF1 patient that was diagnosed as a malignant peripheral nerve sheath tumour, these cells generally show low tumourigenic activity in mice. We found that injection of sNF96.2 cells in subcutaneous tissue or in the sciatic nerve of immunodeficient mice did not result in tumour formation. Given that the cells reliably developed tumours when injected into the brain, we adopted this approach for our *in vivo* model.

To investigate the effects of tranilast in more detail, we established sNF96.2 cells that stably express green fluorescent protein (GFP) and then injected these sNF96.2-GFP cells into the brain of NOD/SCID mice. After 6 weeks, the brain was removed and thin slices of brain tissue were cultured in medium with or without tranilast for up to 5 days. Confocal fluorescence images revealed that tranilast inhibited sNF96.2-GFP cell growth and significantly suppressed cell migration into the brain tissue (Fig. 6b).

### Effects of tranilast on *NF1*-mutated cells derived from clinical specimens

**Figure 7:**
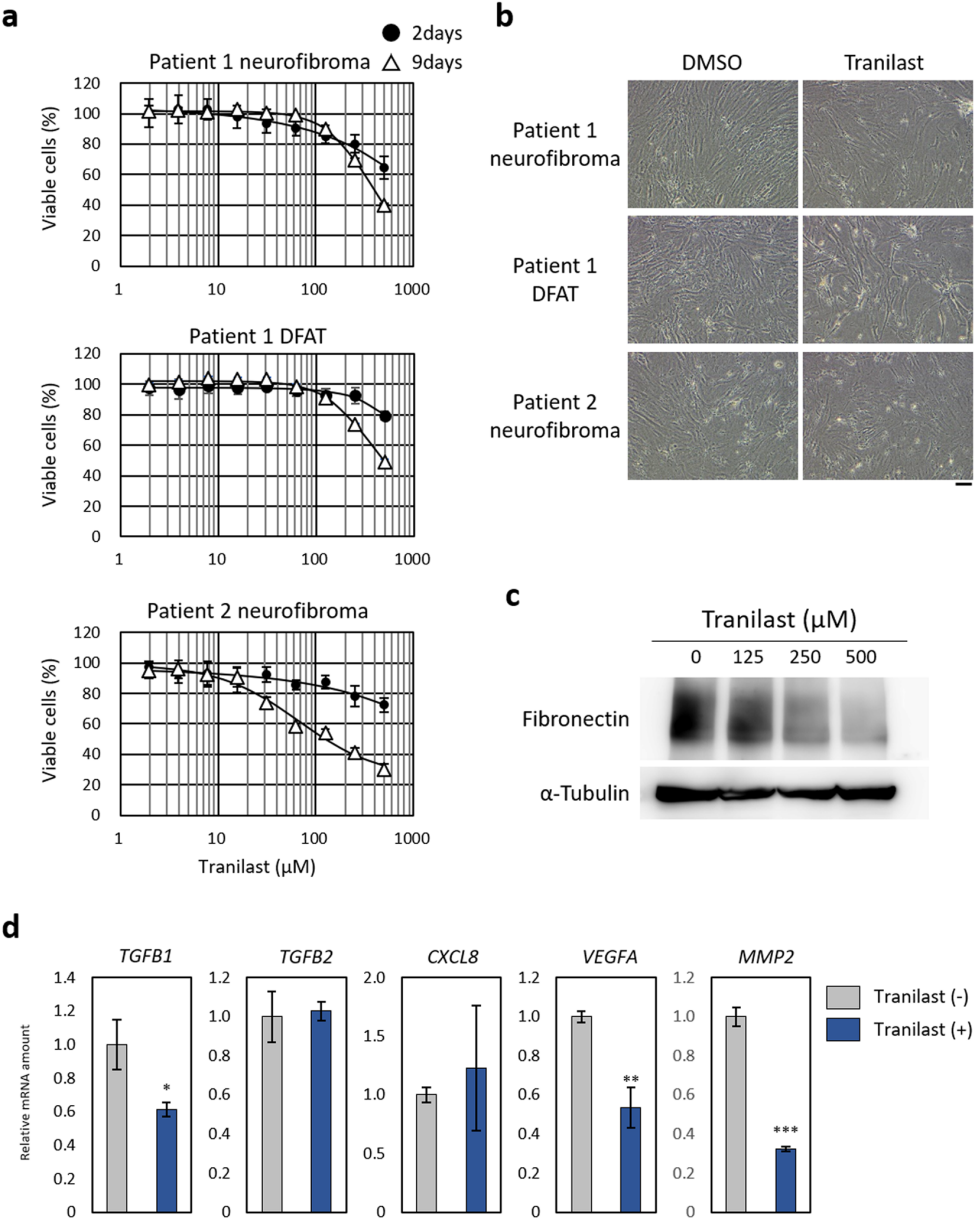
Tranilast suppresses proliferation as well as mesenchymal marker and angiogenesis-related gene expression in cells derived from neurofibromas of NF1 patients. (**a**) Neurofibroma cells or DFAT cells of two patients were cultured in the presence of various concentrations of tranilast for 2 or 9 days, after which cell viability was determined with the CellTiter-Glo assay (Promega). Data are means ± s.d. for six replicates of representative experiments. (**b**) Phase-contrast microscopy of cells incubated with 250 µM tranilast or DMSO vehicle for 9 days as in a. Scale bar, 100 µm. (**c**) Immunoblot analysis of fibronectin in neurofibroma cells of patient 1 that had been treated with the indicated concentrations of tranilast for 2 days. Blots are derived from different regions of different gels. Uncropped images are shown in Supplementary Fig. S6. (**d**) Quantitative RT-PCR analysis of mRNAs for TGF-β1, TGF-β2, IL-8, VEGF-A, and MMP2 in neurofibroma cells of patient 1 that had been incubated with or without 250 µM tranilast for 9 days. Data are means ± s.d. for triplicates from a representative experiment. *P < 0.05, **P < 0.01, ***P < 0.001 versus the corresponding control value (Student’s unpaired *t* test).

With the use of a previously described method^33^, we established neurofibroma cells and dedifferentiated fat (DFAT) cells from neurofibromas of NF1 patients. These cells expressed SOX10, S100, and CD90 (Supplementary Fig. S5), all of which are expressed in Schwann cells. We identified *NF1* mutations in the patients by next-generation sequencing^34^. Peripheral blood specimens from patients 1 and 2 were positive for c.1466A>G, p.Tyr489Cys and c.3213_3214delAA, p.Ser1072Hisfs*16 mutations of *NF1*, respectively. The tumour specimen of patient 1 was also positive for c.6772C>T, p.Arg2258X of *NF1*. Culture of the patient-derived neurofibroma cells and DFAT cells in the presence of various concentrations of tranilast revealed that the drug suppressed the growth of the cells in a concentration-dependent manner (Fig. 7a,b). Immunoblot analysis revealed that tranilast also inhibited the expression of fibronectin in these cells (Fig. 7c), whereas quantitative RT-PCR analysis showed that it significantly attenuated the expression of genes for TGF-β1, VEGF, and MMP2 (Fig. 7d).

### Knockdown of COL3A1 suppresses the proliferation of *NF1*-mutated cells

**Figure 8:**
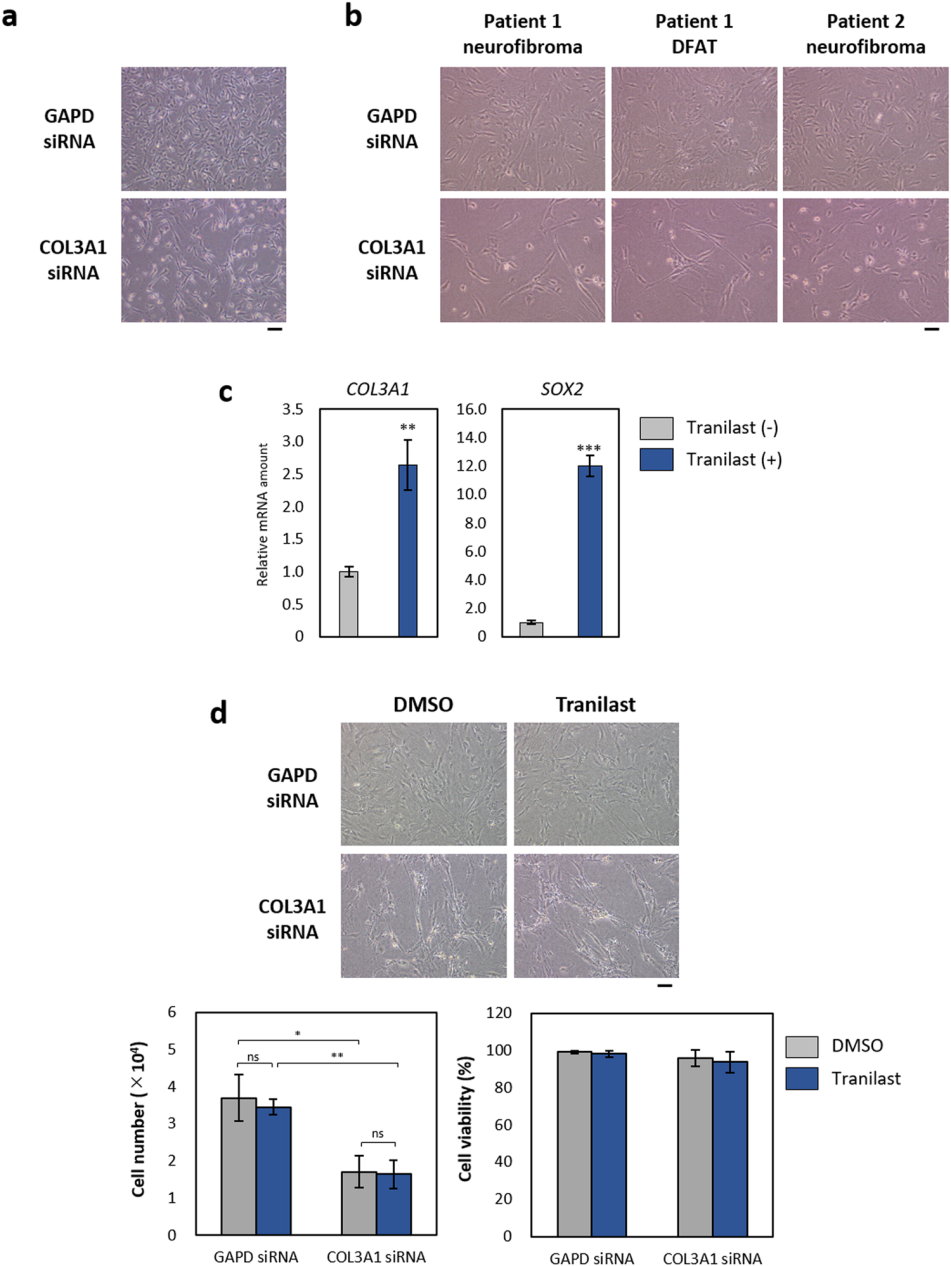
Knockdown of COL3A1 suppresses the proliferation of neurofibromin-deficient cells. (**a**) Phase-contrast microscopy of sNF96.2 cells that had been transfected with control (GAPD) or COL3A1 siRNAs for 2 days. Scale bar, 100 µm. (**b**) Phase-contrast microscopy of neurofibroma cells or DFAT cells from NF1 patients 1 and 2 that had been transfected as in a. Scale bar, 100 µm. (**c**) Quantitative RT-PCR analysis of *COL3A1* and *SOX2* expression in neurofibroma cells of patient 1 that had been exposed to tranilast (250 µM) for 20 days. Data are means ± s.d. for triplicates from a representative experiment. ***P < 0.001 versus corresponding control value (Student’s unpaired *t* test). (**d**) Tranilast-resistant neurofibroma cells derived from patient 1 were transfected with control (GAPD) or COL3A1 siRNAs for 1 day and then exposed to tranilast (250 µM) or DMSO vehicle for 48 h, after which the cells were examined by phase-contrast microscopy. Scale bar, 100 µm. The number of viable cells and the percentage of viable cells were also measured on the basis of trypan blue exclusion. Data are means ± s.d. for triplicates from a representative experiment. *P < 0.05, **P < 0.01 (Student’s unpaired *t* test); ns, not significant.

Both ECM and EMT-TFs are thought to be important in neurofibromas. Neurofibromas comprise various cell types including Schwann cells, fibroblasts, mast cells, and endothelial cells, all of which are embedded in abundant ECM. MMPs regulate the proliferation and infiltration of Schwann cells as well as contribute to the development of peripheral nerve sheath tumours^35–38^. In addition, the EMT-TF Twist has been found to be overexpressed in malignant peripheral nerve sheath tumours, and down-regulation of Twist expression inhibits cell chemotaxis^39,40^. We examined whether collagen type III might affect the proliferation of sNF96.2 cells by depleting the cells of *COL3A1* mRNA by RNA interference. Transfection of the cells with a COL3A1 siRNA inhibited cell growth (Fig. 8a). Such depletion of *COL3A1* also suppressed the growth of neurofibroma cells and DFAT cells derived from NF1 patients (Fig. 8b). These results thus suggested that collagen type III plays a role in maintenance of neurofibromin-deficient cells.

We established the tranilast-resistant neurofibroma cells derived from patient 1 that had been exposed to 250 µM tranilast for 20 days, and we found that the abundance of *COL3A1* and *SOX2* mRNAs was increased in the tranilast-resistant neurofibroma cells (Fig. 8c). Given that SOX2 is a key transcription factor in normal stem cells including pluripotent and tissue-specific stem cells^41^ and that it has been shown to be expressed in brain tumours and other cancers^42^, it is possible that the up-regulation of *COL3A1* and *SOX2* expressions may contribute to the development of resistance to tranilast treatment. We further examined the effect of COL3A1 depletion by siRNA transfection on the number and viability of tranilast-resistant neurofibroma cells from patient 1. The knockdown of COL3A1 markedly suppressed the proliferation of tranilast-resistant neurofibroma cells (Fig. 8d), suggesting that COL3A1 is a major ECM component affecting the proliferation of neurofibromin-deficient cells, however, the depletion of COL3A1 did not increase tranilast sensitivity.

## Discussion

With the use of a cell-based drug screening assay, we have now identified tranilast as an inhibitor of the EMT. We further found that tranilast inhibited the expression of genes related to EMT signalling and angiogenesis in neurofibromin-deficient cells as well as suppressed the proliferation of such cells both *in vitro* and *in vivo*. Moreover, the growth-inhibitory effect of tranilast was more pronounced in neurofibromin-deficient cells than in intact cells. Our data thus suggest that tranilast may inhibit the growth of NF1-associated neurofibromas via suppression of EMT signalling and angiogenesis.

The EMT, in which epithelial cells acquire mesenchymal phenotypes, has been found to occur in various types of cancer, with activation of EMT signalling having been shown to promote cancer progression and metastasis^43^. EMT is associated with increased migratory capacity, invasiveness, resistance to apoptosis, and production of ECM components in cells^44^, and recent studies have indicated that activation of EMT signals also promotes malignancy of nonepithelial tumours^13^. Given that EMT signalling contributes to malignant traits of diverse tumour types, it is a potential therapeutic target. We performed a drug screening assay and thereby identified tranilast as an inhibitor of EMT in the human retinal pigment epithelial cell line ARPE-19. Depletion of neurofibromin by RNA interference in HeLa cells induced EMT signalling, and we found that such signalling was activated in *NF1*-mutated sNF96.2 cells. These results thus suggested that tranilast might suppress the formation or growth of NF1-associated neurofibromas.

Tranilast is an antiallergy drug that is also administered for the treatment of inflammatory diseases, keloids, and hypertrophic scars. It inhibits the release of chemical mediators from mast cells as well as the proliferation of and TGF-β1 and collagen production by fibroblasts^21,45^. In addition, tranilast has been shown to attenuate tumour growth, angiogenesis, migration, invasion, and metastasis through down-regulation of the TGF-β signalling pathway in breast, pancreatic, gastric, and prostate cancer as well as glioma cell lines^46^. TGF-β1 is a key trigger of the EMT program in both normal epithelial cells and cancer cells. In the present study, we determined the effects of tranilast on neurofibromin-deficient cells.

We found that tranilast suppressed the expression of mesenchymal markers in *NF1*-mutated sNF96.2 cells as well as in neurofibroma cells from NF1 patients. The abundance of mRNAs for various EMT-TFs, collagens, hyaluronan synthases, and integrins was also down-regulated by tranilast in sNF96.2 cells, suggesting that tranilast suppresses the mesenchymal characteristics of these cells. We also determined that tranilast suppressed the proliferation of both sNF96.2 cells and NF1 patient–derived cells, and that such growth suppression was more effective in HeLa and NIH3T3 cells depleted of neurofibromin than in intact cells. These results indicate that tranilast suppresses EMT signalling that is induced by neurofibromin deficiency and which gives rise to neurofibroma growth.

We detected the expression of collagen type III, an EMT-related ECM component, in neurofibroma specimens from NF1 patients. The expression of collagen type III in sNF96.2 cells was down-regulated at both the mRNA and protein levels by treatment with tranilast. Furthermore, we found that targeting of the collagen type III gene *COL3A1* by RNA interference induced growth suppression both in sNF96.2 cells and in NF1 patient–derived cells. The expression of *COL3A1* has previously been implicated in promotion of cell proliferation, metastasis, and invasion^47–49^. Tranilast may suppress the proliferation of neurofibromin-deficient cells by down-regulating mesenchymal phenotypes such as the production of ECM components. TGF-β1 induces transcriptional activation of *COL3A1* in renal fibroblast cell lines^50^. In addition, tranilast was recently shown to suppress transcription of *COL3A1* and *CDK2* through regulation of the microRNA miR-29c in leiomyoma smooth muscle cells^51^. Tranilast might therefore have therapeutic potential for various benign tumours.

The expression of VEGF-A was found to be increased in cells undergoing TGF-β–induced EMT, and the extent of vascularization in tumours formed by MTΔECad mesenchymal cells correlated with the level of VEGF-A expression^52^, suggestive of a relation between EMT and tumour angiogenesis. We found that tranilast inhibited expression of the angiogenesis-related genes for TGF-β, IL-8, VEGF-A, and MMP2 in sNF96.2 cells. All of these angiogenic factors have been associated with tumour angiogenesis^29–32^. Tranilast may therefore inhibit the formation of tumour blood vessels by suppressing the expression of angiogenesis-related genes, with such activity possibly contributing to its antitumour effects. Angiogenesis has been shown to accompany the progression of NF1-associated neurofibromas^53^. We found that expression of the genes for TGF-β, IL-8, VEGF, and MMP2 was increased in *NF1*-mutated sNF96.2 cells compared with normal HSCs. However, transient depletion of neurofibromin by siRNA transfection did not increase the expression of these genes in HSCs, suggesting that chronic deficiency of neurofibromin is indirectly associated with angiogenesis. Angiogenesis is a potential therapeutic target for cancer. Many angiogenesis inhibitors, including bevacizumab, aflibercept, and ramucirumab, are thus administered as anticancer agents, and the development of new antiangiogenesis drugs is being actively pursued^54–60^. Such agents may prove effective for inhibition of neurofibroma growth. Our data also suggest that tranilast may inhibit vascularization, although further studies are required to confirm this possibility.

Finally, we found that the expression of *COL3A1* and *SOX2* was increased in tranilast-resistant neurofibroma cells, suggesting that the encoded proteins may give rise to resistance to tranilast treatment. The pluripotency-associated transcription factor SOX2 was recently shown to be expressed in a cell population manifesting properties of cancer stem cells or tumour-initiating cells and to be associated with drug resistance^42^. Attenuation of the expression of *SOX2* or *COL3A1* is therefore a potential approach to circumventing resistance to tranilast treatment. Inhibition of SOX2 expression has been shown to suppress cancer initiation and tumour cell proliferation, migration, invasion, and metastasis as well as to induce apoptosis in oligodendroglioma, lung cancer, breast cancer, and osteosarcoma cells^61–64^. Combined treatment with a SOX2 blocker and tranilast is therefore also a potential approach to inhibition of neurofibroma growth.

In summary, tranilast inhibited the expression of EMT- and angiogenesis-related genes associated with neurofibromin deficiency as well as suppressed the proliferation of neurofibromin-deficient cells both *in vitro* and *in vivo*. Our findings thus suggest that tranilast and other EMT inhibitors warrant further investigation as potential therapeutic agents for NF1-associated neurofibromas.

## Methods

### Cell lines and cell culture

ARPE-19 cells were obtained from American Type Culture Collection (ATCC) and were maintained in Dulbecco’s modified Eagle’s medium (DMEM)–F-12 (Sigma) supplemented with 10% fetal bovine serum (FBS), penicillin (100 U/ml), and streptomycin (100 µg/ml). sNF96.2, HeLa, and NIH3T3 cells were also obtained from ATCC and were maintained in DMEM supplemented with 10% FBS. HSCs were obtained from ScienCell Research Laboratories and were cultured in Schwann cell medium (SCM, ScienCell Research Laboratories).

### Patients

Patients who met the NIH clinical diagnostic criteria for NF1 (Neurofibromatosis Conference Statement, 1988) were recruited. Tissue samples were obtained during tumour resection surgery at Keio University Hospital. Whole-blood specimens were also obtained for gene analysis. All patients provided written informed consent, and this aspect of the study was approved by the institutional review board of Keio University.

### Preparation of tumour cells from NF1 patients

Neurofibroma cells and DFAT cells were established from neurofibromas of NF1 patients as described previously^33^. Tumor tissue was washed extensively with phosphate-buffered saline (PBS), minced in DMEM containing 0.1% collagenase (Sigma) and 2% bovine serum albumin (BSA, Sigma), and dissociated by incubation for 1 h at 37 °C with gentle shaking. The cell suspension was passed through a 100-µm cell strainer (Corning) to remove tissue debris. The cells were then washed three times with PBS by centrifugation at 135 × *g* for 3 min. The neurofibroma cells at the bottom of the tube and the floating stromal adipocytes were collected separately and purified by centrifugation three times. For establishment of DFAT cells, the stromal adipocytes were placed in a culture flask (Corning) filled with DMEM supplemented with 20% FBS, which generated a sealed environment, and the flask was then inverted and incubated at 37 °C in a humidified atmosphere of 5% CO_2_. Under this culture condition, the stromal adipocytes float up through the medium and adhere to the top inner surface (ceiling) of the flask. After 1 week, the cells were firmly attached to the ceiling and had transformed into fibroblast-like DFAT cells with no visible fat droplets. Both the neurofibroma cells and the DFAT cells were maintained in DMEM supplemented with 20% FBS at 37 °C in a humidified atmosphere of 5% CO_2_.

### Focus formation assay

ARPE-19 cells were cultured in 96-well plates for 5 days and then exposed for 2 days to TNF-α (100 ng/ml, eBioscience) and TGF-β2 (5 ng/ml, eBioscience) in the absence or presence of SB431542 (10 µM, Calbiochem) or test drugs. They were then fixed for 30 min at room temperature with 4% paraformaldehyde in PBS, washed with PBS, incubated for 60 min at room temperature with Hoechst 33342 (Invitrogen) and phalloidin–Alexa Fluor 568 (Invitrogen) in PBS, and washed again with PBS. The cells were examined with a high-throughput image screening system (ImageXpress ULTRA, Molecular Devices) for quantitation of focus formation, with a focus being defined as a cell aggregate with an area of Hoechst 33342 fluorescence greater than a certain threshold. The fluorescence intensity of each focus was measured, and the percentage inhibition of focus formation by test drugs was determined as: 100 – [100 × (fluorescence intensity for all foci in the presence of the test drug)/(fluorescence intensity for all foci in the absence of the drug)]. The median inhibitory concentration for each drug was determined from the concentration-response curve.

### RNA interference

NF1, COL3A1, and negative control (GAPD) siRNAs were obtained from Dharmacon and were introduced into cells by transfection with the Lipofectamine RNAiMAX reagent (Invitrogen). NIH3T3 cells were transfected with plasmids for NF1 shRNAs (pSUPER-NF1 249, 5′-GATCCCCCAAGGAGTGTCTGATCAACTTCAAGAGAGTTGATCAGACACTCCTTGTTTTTA-3′ and 5′-AGCTTAAAAACAAGGAGTGTCTGATCAACTCTCTTGAAGTTGATCAGACACTCCTTGGGG-3′; pSUPER-NF1 611, 5′-GATCCCCGGTTACAGGAGTTGACTGTTTCAAGAGAACAGTCAACTCCTGTAACCTTTTTA-3′ and 5′-AGCTTAAAAAGGTTACAGGAGTTGACTGTTCTCTTGAAACAGTCAACTCCTGTAACCGGG-3′) or a control plasmid with the use of the Lipofectamine 2000 transfection reagent (Thermo Fisher Scientific), and the cells were then subjected to selection with puromycin (3 µg/ml).

### Quantitative RT-PCR analysis

Total RNA was extracted from cells with the use of an RNeasy Mini Kit (Qiagen), and portions (1 μg) of the isolated RNA were subjected to RT with a Transcriptor first-strand cDNA synthesis kit (Roche). The resulting cDNA was subjected to real-time PCR analysis with SYBR Premix ExTaq (TaKaRa) and a Thermal Cycler Dice RealTime System (TP800, TaKaRa). Primer sequences are listed in Supplementary Table S1. The amplification protocol comprised an initial incubation at 95 °C for 2 min followed by 40 cycles of 95 °C for 30 s and 60 °C for 30 s. The abundance of each target mRNA was normalized by that of hypoxanthine phosphoribosyltransferase 1 (HPRT1) mRNA.

### Immunoblot analysis

Cells were collected and lysed in lysis buffer (2% SDS, 10% glycerol, 50 mM Tris-HCl (pH 6.8), 100 mM dithiothreitol). Equal amounts of total lysates were fractionated by SDS-polyacrylamide gel electrophoresis, and the separated proteins were transferred to a nitrocellulose filter and probed with primary antibodies to fibronectin (Santa Cruz Biotechnology), to N-cadherin (Santa Cruz Biotechnology), to collagen type I (Abcam), or to α-tubulin (Sigma-Aldrich). Immune complexes were detected with horseradish peroxidase–conjugated secondary antibodies (GE Healthcare and Dako), enhanced chemiluminescence reagents (ImmunoStar LD, Wako), and an LAS-3000mini instrument (GE Healthcare).

### Fluorescence or immunofluorescence analysis

ARPE-19 cells cultured in 96-well plates were fixed for 30 min at room temperature with 4% paraformaldehyde in PBS, washed with PBS, and exposed for 1 h at room temperature to 3% BSA in PBS before incubation overnight at 4 °C with biotinylated HABP (5 µg/ml, Seikagaku) and 3% BSA in PBS. The cells were then washed with PBS, incubated for 60 min at room temperature with streptavidin–Alexa Fluor 488 (Invitrogen) and Hoechst 33342 in PBS containing 3% BSA, washed again with PBS, and imaged with a BZ-9000 fluorescence microscope (Keyence).

sNF96.2 cells cultured in 35-mm dishes were fixed for 15 min at room temperature with 4% paraformaldehyde in PBS, washed with PBS, and then permeabilized for 30 min at room temperature with 0.2% Triton X-100 in PBS. The cells were stained for 90 min at room temperature with antibodies to collagen type III (Abcam) diluted in PBS containing 1% BSA, washed with 0.2% Triton X-100 in PBS, incubated for 60 min at room temperature with Alexa Fluor 594–conjugated secondary antibodies (Invitrogen) in PBS containing 1% BSA, and washed again with 0.2% Triton X-100 in PBS. They were finally stained for 5 min with Hoechst 33342 in PBS containing 1% BSA, washed with 0.2% Triton X-100 in PBS, and imaged with a BZ-9000 fluorescence microscope (Keyence).

### Cell viability assay

Cells were seeded at a density of 2.5 × 10^3^ per well in 96-well plates and incubated for 24 h before exposure to various concentrations of tranilast (Calbiochem) for the indicated times. Cell viability was then assayed with a CellTiter-Glo Kit (Promega) and an EnVision Plate Reader (PerkinElmer).

### Cell transplantation

sNF96.2 cells (1 × 10^6^ cells in 2 µl of Hanks’ balanced salt solution) were injected as a single-cell suspension into the brain of 6-week-old female NOD/SCID mice (Charles River) that had been anesthetized by exposure to 1% to 3% isoflurane. After 3 weeks, the mice were treated by oral administration of tranilast (300 mg/kg) or vehicle (1% NaHCO_3_) once a day for 8 weeks. This aspect of the study was approved by the Animal Care and Use Committee of Keio University School of Medicine.

### Tissue slice culture

Brain tissue slice culture was performed as previously described^65^. sNF96.2-GFP cells were injected into the brain of NOD/SCID mice as described above. After 6 weeks, the brain was removed and thin tissue slices were cultured in the presence of tranilast (750 µM) or DMSO vehicle. The slices were imaged with a Fluoview FV10i microscope (Olympus). The maximum tumour diameter and the maximum length of the invasion path were determined from the images.

### Immunohistochemistry

Immunohistochemical staining was performed as described previously^14^ with antibodies to vimentin (Dako), to collagen type I (Abcam), to collagen type III (Abcam), to ZEB1 (Santa Cruz Biotechnology), or to SOX2 (R&D Systems).

### Collagen type III ELISA

The collagen type III content of sNF96.2 cells was determined with the use of a Human Collagen Type III ELISA kit (Kamiya Biomedical Co.).

### Statistical analysis

Data are presented as means ± s.d. and were analysed with Student’s unpaired *t* test. A *P* value of <0.05 was considered statistically significant.

### Data availability

Representative data are provided in this published article and its Supplementary Information files. Other data sets generated during and/or analysed during the current study are available from the corresponding author on reasonable request.

All methods were performed in accordance with the relevant guidelines and regulations.

## Electronic supplementary material

Supplementary Information
