# Supplementary material for: Tranilast inhibits the expression of genes related to epithelial-mesenchymal transition and angiogenesis in neurofibromin-deficient cells
Source: Sci Rep. 2018 Apr 17;8:6069. doi: 10.1038/s41598-018-24484-y (PMC5904101; doi:10.1038/s41598-018-24484-y)

Tranilast inhibits the expression of genes related to epithelial-mesenchymal transition and angiogenesis in neurofibromin-deficient cells

Ritsuko Harigai[1], Shigeki Sakai[2], Hiroyuki Nobusue[1], Chikako Hirose[1,3], Oltea Sampetrean[1], Noriaki Minami[1,4], Yukie Hata[5], Takashi Kasama[5], Takanori Hirose[6], Toshiki Takenouchi[7], Kenjiro Kosaki[8], Kazuo Kishi[2], Hideyuki Saya[1] &Yoshimi Arima[1]*

[1]Division of Gene Regulation, Institute for Advanced Medical Research, Keio University School of Medicine, Tokyo 160-8582, Japan. [2]Department of Plastic and Reconstructive Surgery, Keio University School of Medicine, Tokyo 160-8582, Japan. [3]Department of Surgery, Keio University School of Medicine, Tokyo 160-8582, Japan. [4]Department of Neurosurgery, Kobe University Graduate School of Medicine, Hyogo 650-0017, Japan. [5]Department of Biomedical Research & Development, Link Genomics Inc., Tokyo 103-0024, Japan. [6]Department of Pathology for Regional Communication, Kobe University Graduate School of Medicine, Hyogo, 650-0017, Japan. [7]Department of Paediatrics, Keio University School of Medicine, Tokyo 160-8582, Japan. [8]Center for Medical Genetics, Keio University School of Medicine, Tokyo 160-8582, Japan.

*Address correspondence and requests for materials to Y.A. (email: arima@z7.keio.jp).

Supplementary Information

Supplementary Figure S1. Knockdown of neurofibromin induces EMT-like changes in HeLa cells. **a**, Phase-contrast microscopy of HeLa cells transfected with control (GAPD) or neurofibromin (NF1) siRNAs for 48 h. Scale bar, 100 µm. **b**, Quantitative RT-PCR analysis of *NF1*, *FN1* (fibronectin), *CDH2* (N-cadherin), *SNAI1* (Snail), *SNAI2* (Slug), *TWIST1*, and *ZEB1* mRNAs in HeLa cells transfected with NF1 or control (GAPD) siRNAs for 48 h. Data are expressed relative to the corresponding value for cells transfected with the GAPD siRNA and are means ± s.d. for triplicates from a representative experiment. *$P < 0.05$, **$P < 0.01$, ***$P < 0.001$ versus corresponding control value (Student's unpaired *t* test).

Supplementary Figure S2. Tranilast suppresses mesenchymal marker gene expression in sNF96.2 cells. sNF96.2 cells were incubated in the absence or presence of tranilast (250 µM) for 48 h, after which the relative abundance of mRNAs encoding the indicated EMT-TFs (**a**), collagens (**b**), hyaluronan synthases (**c**), and integrins (**d**) was determined by quantitative RT-PCR analysis. Data are means ± s.d. for triplicates from a representative experiment. *$P < 0.05$, **$P < 0.01$, ***$P < 0.001$ versus corresponding control (Student's unpaired *t* test).

Supplementary Figure S3. Expression of mesenchymal markers and SOX2 in NF1-associated neurofibromas. Subcutaneous tissue from NF1 patients with neurofibromas (patients 3 and 4) was subjected to hematoxylin-eosin (HE) staining as well as to immunohistochemical analysis of collagen types I and III, vimentin, and ZEB1 (**a**) or of SOX2 (**b**). Scale bars, 100 µm.

Supplementary Figure S4. Expression of angiogenesis-related genes is increased in sNF96.2 cells compared with HSCs but is not increased by transient knockdown of neurofibromin in HSCs. sNF96.2 cells and HSCs (**a**) as well as HSCs transfected with control (GAPD) or

neurofibromin (NF1) siRNAs for 24 h (**b**) were subjected to quantitative RT-PCR analysis of the indicated mRNAs. Data are means ± s.d. for duplicates from a representative experiment. $*P < 0.05$, $**P < 0.01$, $***P < 0.001$ (Student's unpaired *t* test) versus corresponding value for HSCs (**a**) or control siRNA–transfected cells (**b**).

Supplementary Figure S5. Flow cytometric analysis of SOX10, S100, CD90, and CD31 expression in neurofibroma cells and DFAT cells derived from NF1 patients. Single-cell suspensions in PBS were labeled with Alexa Fluor 647–conjugated mouse monoclonal antibodies to human CD90 or CD31 (BioLegend). For staining of SOX10 or S100, the cells were fixed and permeabilised with BD Cytofix/Cytoperm Fixation/Permeabilization solution (BD Biosciences), and then labeled with rabbit monoclonal antibodies to human SOX10 (Abcam) or rabbit polyclonal antibodies to human S100 (Dako). Immune complexes containing the rabbit antibodies were detected with Alexa Fluor 633–conjugated goat antibodies to rabbit immunoglobulin G (Invitrogen). The cells were analysed with the Attune Acoustic Focusing Cytometer (Invitrogen). The percentages of cells positive for SOX10, S100, CD90, or CD31 are indicated. SOX10, S100, and CD90 are expressed in Schwann cells, and CD31 is an endothelial cell marker.

Supplementary Figure S6. Uncropped images for the blots shown in Figures 2a and 7c.

Supplementary Table S1. Primers for quantitative RT-PCR analysis.

Gene	Orientation	Sequence
TGFB1	Forward	5′-CATCGTGTACTACGTGGGC-3′
	Reverse	5′-GGAGCGCACGATCATGTTG-3′
TGFB2	Forward	5′-GAAGCATCTGCTTCTCCTTGC-3′
	Reverse	5′-GGGTGTTTTGCCAATGTAGTAGAG-3′
CXCL8	Forward	5′-GAATGGGTTTGCTAGAATGTGATA-3′
	Reverse	5′-CAGACTAGGGTTGCCAGATTTAAC-3′
VEGFA	Forward	5′-TCCCTCTTGGAATTGGATTCG-3′
	Reverse	5′-GTATGTGGGTGGGTGTGTCTACAG-3′
MMP2	Forward	5′-CCAAGACCCTGGGGCCCTGAT-3′
	Reverse	5′- GGCCCCATAGAGCTCCTGAATGC-3′
FN1	Forward	5′-CCAGTCCTACAACCAGTATTCTC-3′
	Reverse	5′-CTTCTCTGTCAGCCTGTACATC-3′
CDH2	Forward	5′-CATCAAGCCTGTGGGAATCC-3′
	Reverse	5′-AATGAAGTCCCCAATGTCTCCAG-3′
COL1A1	Forward	5′-GTCACTGTCGATGGCTGC-3′
	Reverse	5′-CGTCGAAGCCGAATTCCTG-3′
SNAI1	Forward	5′-CCTCAAGATGCACATCCGAAG-3′
	Reverse	5′-ACATGGCCTTGTAGCAGCCA-3′
SNAI2	Forward	5′-CCCACACATTACCTTGTGTTTGCAA-3′
	Reverse	5′-CAAATGCTCTGTTGCAGTGAGG-3′
TWIST1	Forward	5′-GGACAAGCTGAGCAAGATTCAGA-3′
	Reverse	5′-GTGAGCCACATAGCTGCAG-3′
ZEB1	Forward	5'-GGCAGAGAATGAGGGAGAAG-3′

	Reverse	5′-CTTCAGACACTTGCTCACTACTC-3′
ZEB2	Forward	5′-GTGACAAGACATTCCAGAAAAGCAG-3′
	Reverse	5′-GAGTGAAGCCTTGAGTGCTC-3′
COL3A1	Forward	5′-CACGCAAGGCTGTGAGAC-3′
	Reverse	5′-CAGGGCCAACGTCCAC-3′
COL4A1	Forward	5′-GGGACCTGCAATTACTACGC-3′
	Reverse	5′-CTCATACAGACTTGGCAGCG-3′
COL4A2	Forward	5′-CTGGCTGACCACCATTCC-3′
	Reverse	5′-CATGCACACCTGGCAGC-3′
COL6A3	Forward	5′-GACGAAGGAACTTGCAGGG-3′
	Reverse	5′-GTTTGGCGAGCACAGGAG-3′
HAS1	Forward	5′-CAGCGTAGCACACGAGG-3′
	Reverse	5′-CCAGTACAGCGTCAACATGG-3′
HAS2	Forward	5′-GGAACGTTGCTCTATGCATGC-3′
	Reverse	5′-CCGCCTGCCACACTTATTGAT-3′
HAS3	Forward	5′-CGGTGGACTACATCCAGG-3′
	Reverse	5′-CCTCCAGGACTCGAAGCAT-3′
ITGA1	Forward	5′-GGATCTTTTCCAATGCCAGAG-3′
	Reverse	5′-ATCCAGTTGGGTACAGCACAG-3′
ITGA2	Forward	5′-GGAAAGTGCATACAACACTGGA-3′
	Reverse	5′-GCATGTTACTTCTGTCCCATCA-3′
ITGA3	Forward	5′-TACTCTTTACCTTTGCGGATGC-3′
	Reverse	5′-ACTCCTTCTGGAACTGGACCTC-3′
ITGA5	Forward	5′-CTCAGGCCAGCCCTACATTA-3′
	Reverse	5′-TTCTGCTCCCCAAACACTTC-3′

ITGA6	Forward	5′-AGCCTTCATTGATGTGACTGC-3′
	Reverse	5′-CCCAGCGAGAATAGCCACTA-3′
ITGA7	Forward	5′-TCCCCAGCAACTCTTCTTCTC-3′
	Reverse	5′-AACCGTGACCTCATACTTGACC-3′
ITGA10	Forward	5′-TCCCCTTCTCAAAGGATTGTG-3′
	Reverse	5′-CTGGAGCCTCTGATGTCCATA-3′
ITGA11	Forward	5′-CCAGTGACCTTCTCAGTCGAGTA-3′
	Reverse	5′-AACACAAGGTCAGGGACACAG-3′
ITGAV	Forward	5′-GTCTGTAAACCCAAGCTGGAAG-3′
	Reverse	5′-CAGTGGAATGGAAACGATGAG-3′
ITGB1	Forward	5′-TCATGACAGAAGGGAGTTTGC-3′
	Reverse	5′-ACCACAGTTGTTACGGCACTC-3′
ITGB4	Forward	5′-TTCCTAGTGGATGGGCTGAC-3′
	Reverse	5′-CATGTGGGTGCTAAGGGTTC-3′
NF1	Forward	5′-CTTAGTATCACTGCCAACCTTAAC-3′
	Reverse	5′-GGAATGCTGGGAAGTTGCAAGT-3′
TNFA	Forward	5′-GAGGCCAAGCCCTGGTATG-3′
	Reverse	5′-GTAGACCTGCCCAGACTC-3′
IL1B	Forward	5′-GAGTCTGCCCAGTTCCC-3′
	Reverse	5′-CAGTTATATCCTGGCCGCC-3′
IL6	Forward	5′-CTGACCCAACCACAAATGCC-3′
	Reverse	5′-CATTTGCCGAAGAGCCCTC-3′
KITLG	Forward	5′-AGCCAAGTCTTACAAGGGCA-3′
	Reverse	5′-TAAATGAGACCCAAGTCCCG-3′
FGF2	Forward	5′-CCAGTTGGTATGTGGCACTG-3′

	Reverse	5′-CAGCTCTTAGCAGACATTGG-3′
MMP1	Forward	5′-TGGCCACAACTGCCAAATGGG-3′
	Reverse	5′-GCACATTCTGTCCCTGAACAGCCC-3′
MMP13	Forward	5′-GACTTCCCAGGAATTGGTG-3′
	Reverse	5′-GCTGGCATGACGCGAAC-3′
CD44	Forward	5′-GAGAGGCCAGCAAGTCTC-3′
	Reverse	5′-GTCCACATTCTGCAGGTTCC-3′
HPRT1	Forward	5′-CCTGGCGTCGTGATTAGTGA-3′
	Reverse	5′-TCGAGCAAGACGTTCAGTCC-3′

Supplemental Fig.1

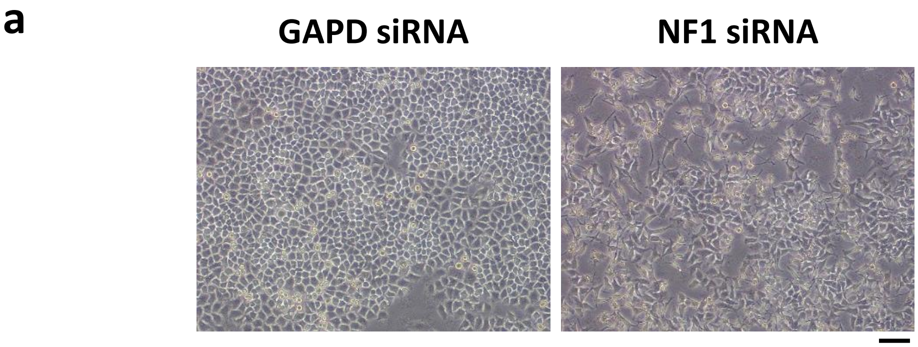

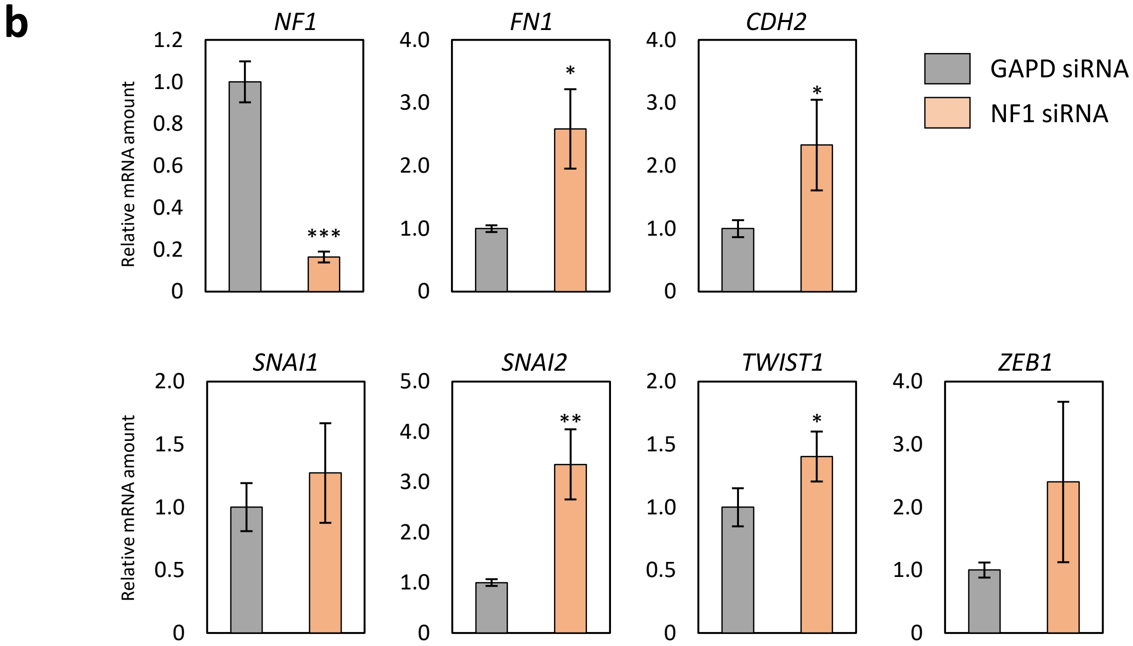

Supplemental Fig.2

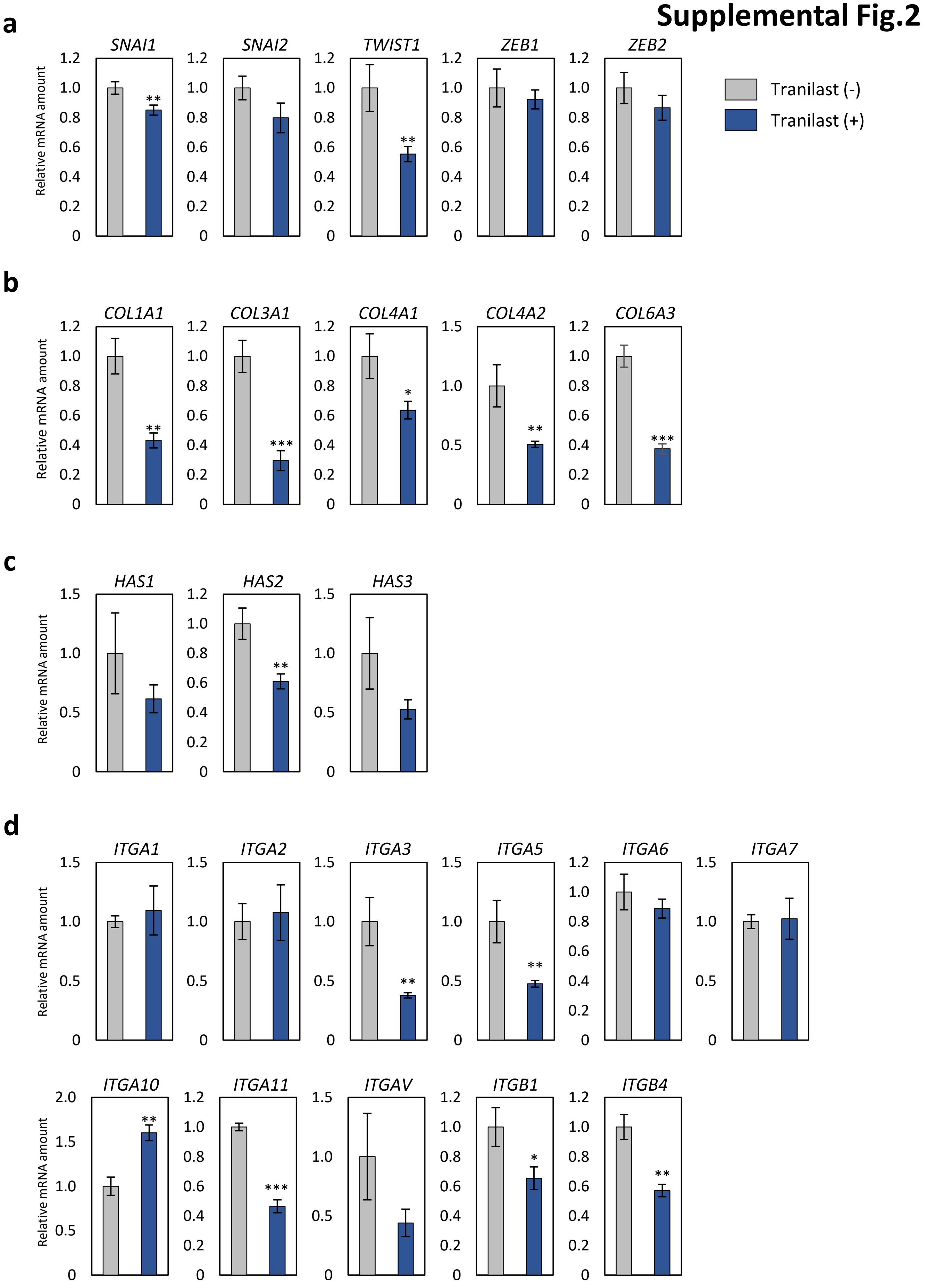

Supplemental Fig.3

a

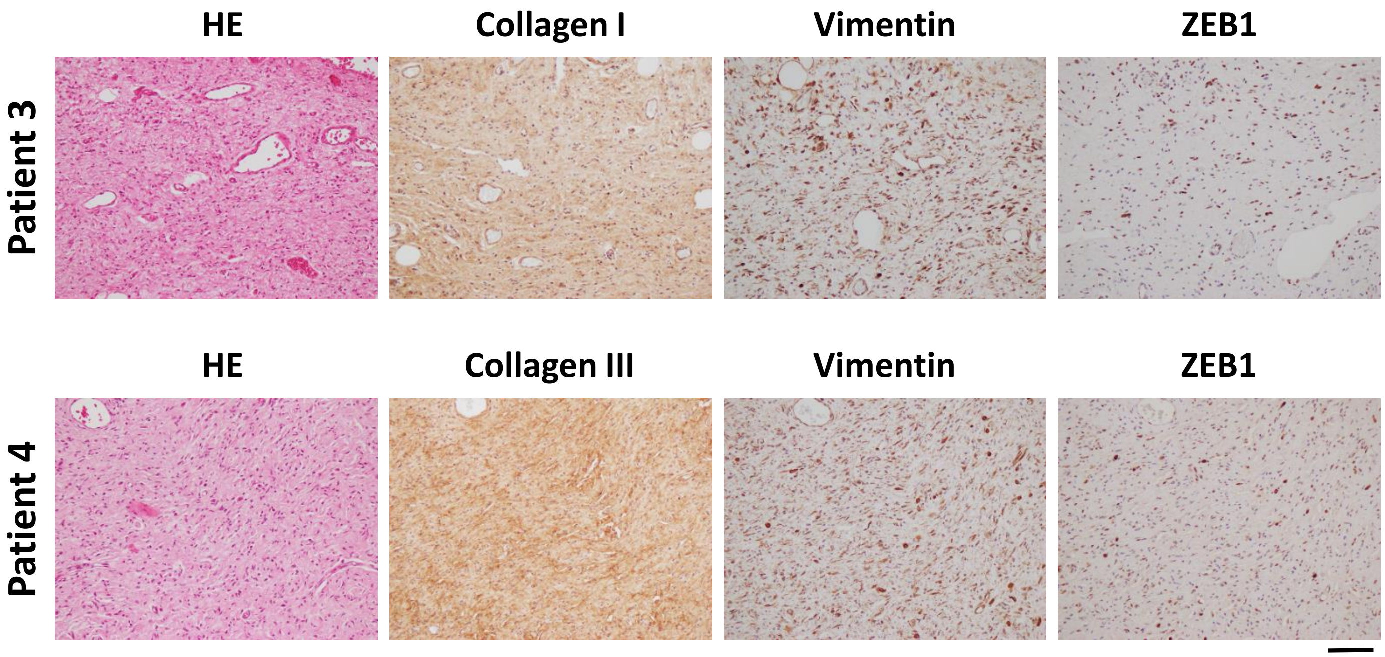

b

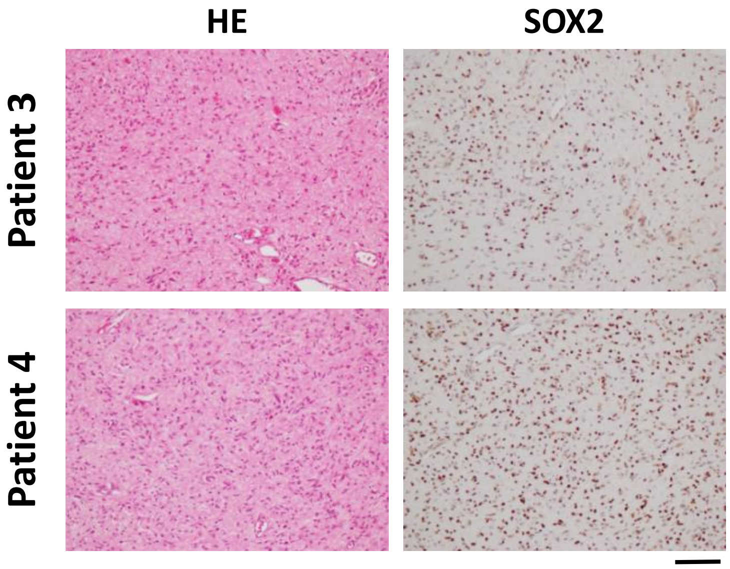

Supplemental Fig.4

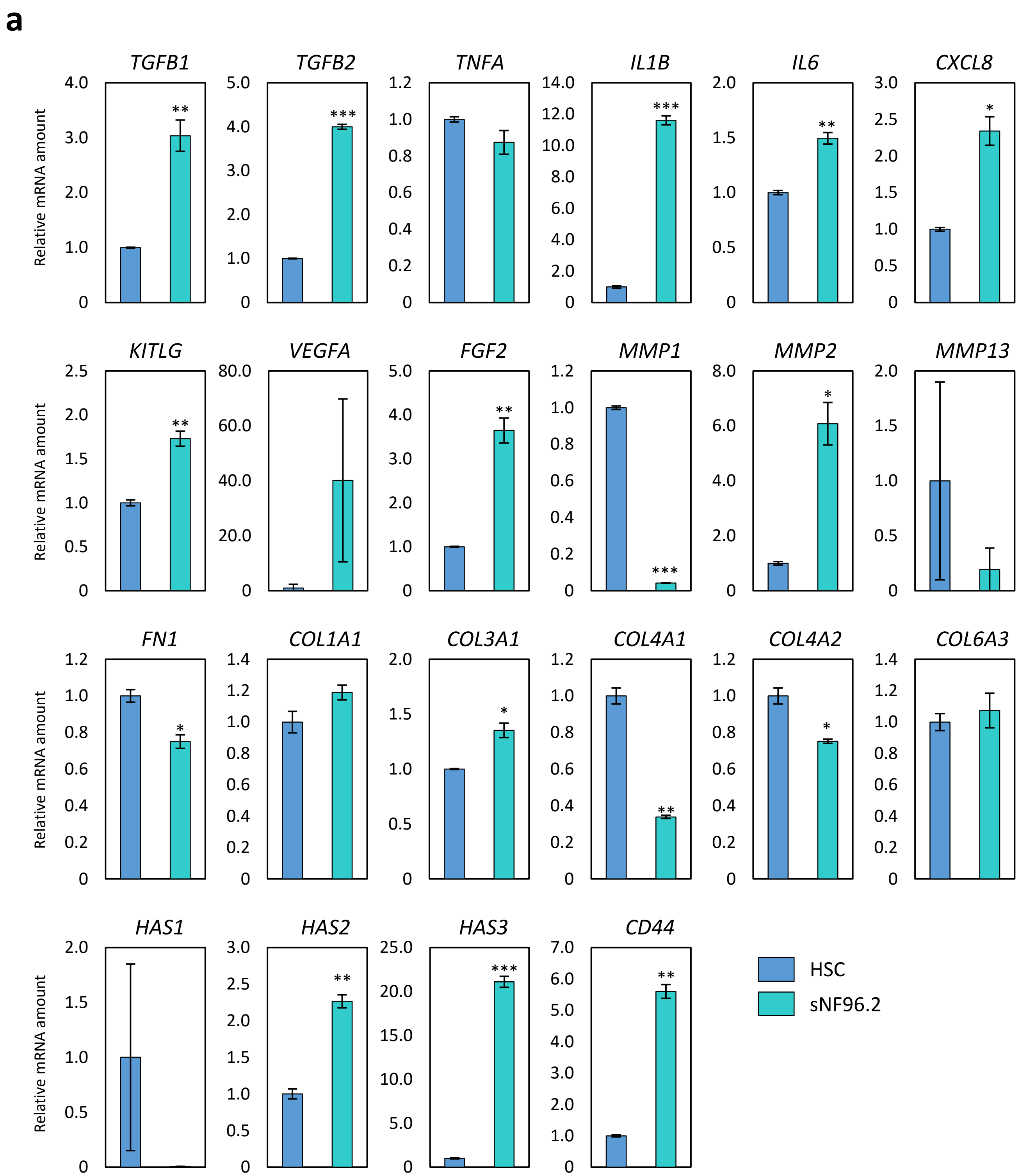

Supplemental Fig.4

b

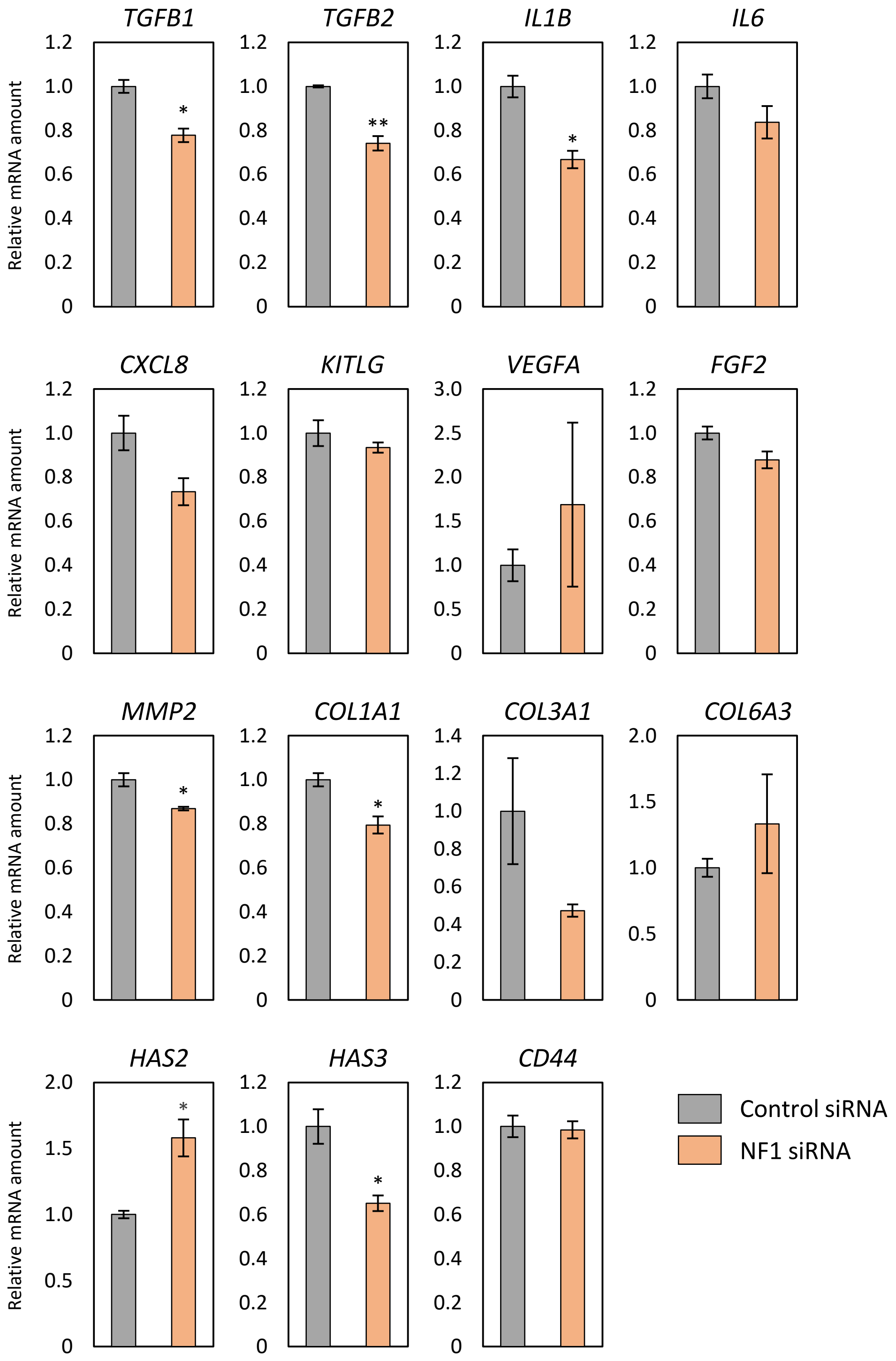

Supplemental Fig.5

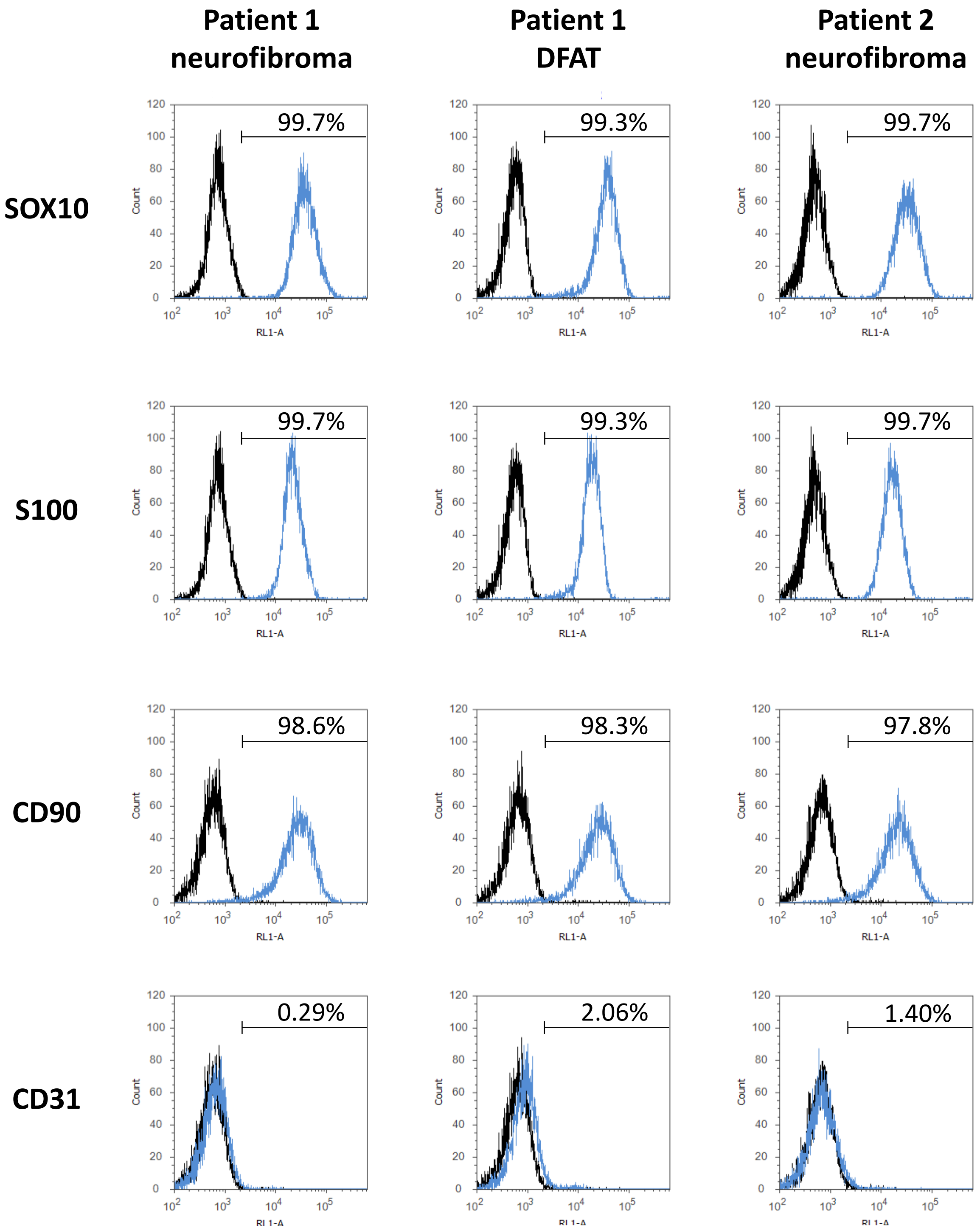

Supplemental Fig.6

Figure 2a. First panel original figure.

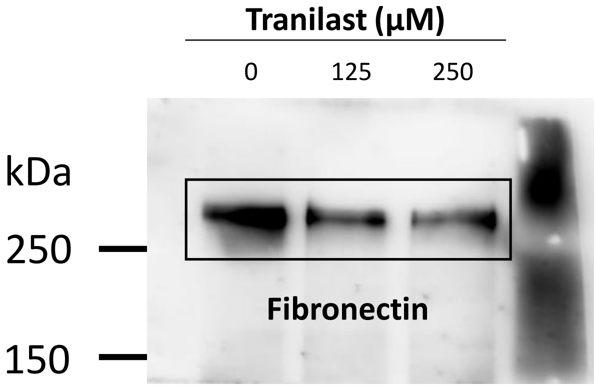

Figure 2a. Second panel original figure.

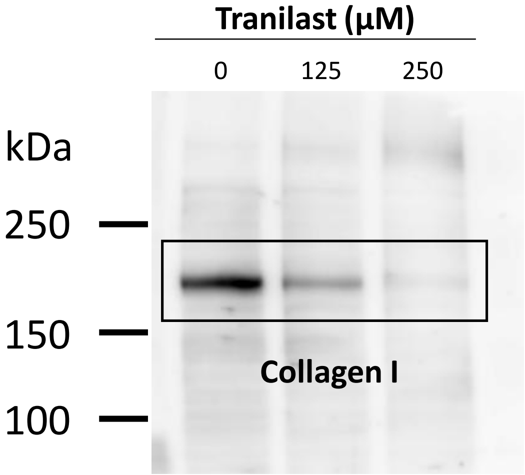

Figure 2a. Third panel original figure.

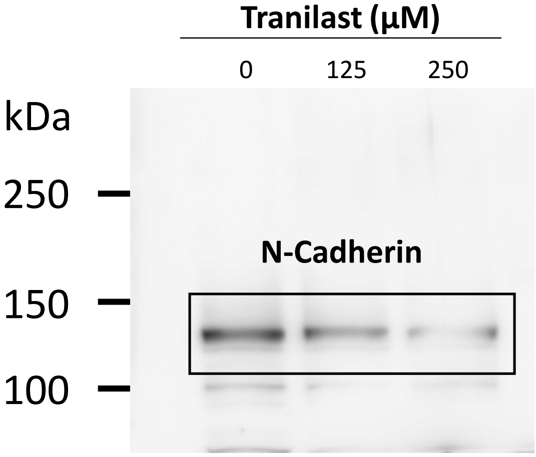

Figure 2a. Fourth panel original figure.

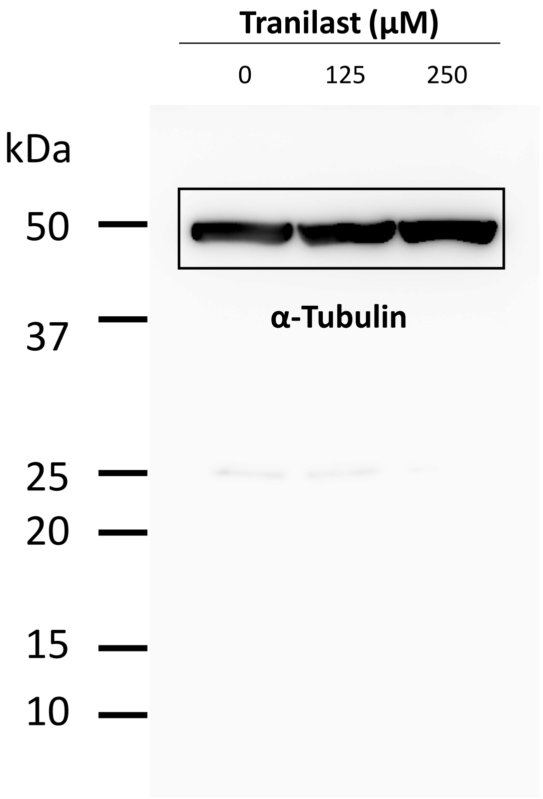

Figure 7c. Top panel original figure.

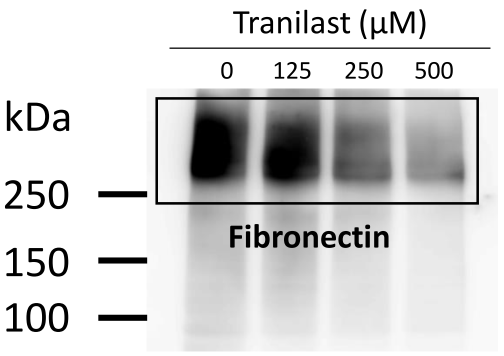

Figure 7c. Bottom panel original figure.

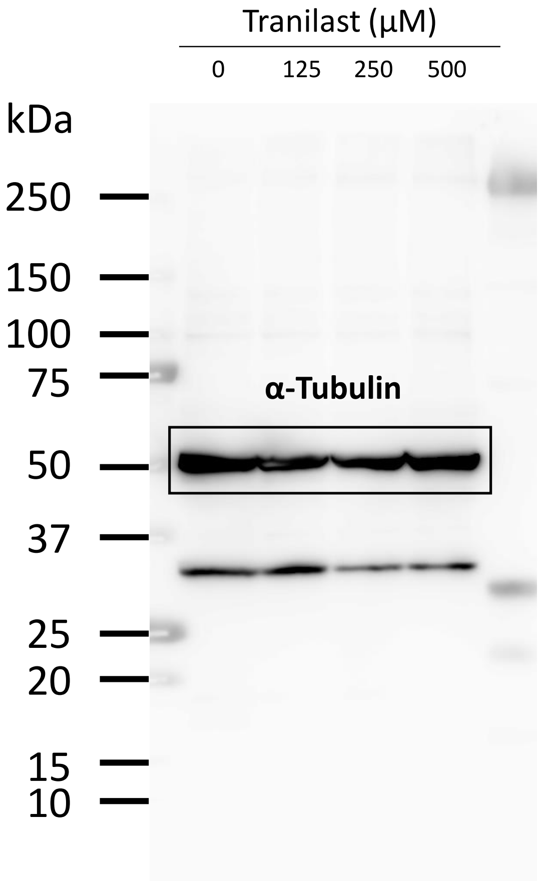

Supplement: Supplementary file 1 — Supplementary Information [file 41598_2018_24484_MOESM1_ESM.pdf]
